# In silico identification of novel natural compounds as potential KIFC1 inhibitors for the therapeutic intervention of triple-negative breast cancer

**DOI:** 10.3389/fbinf.2025.1689172

**Published:** 2025-12-16

**Authors:** Prashant Kumar Tiwari, Mukesh Kumar, Richa Mishra, Xiaomeng Zhang, Sanjay Kumar

**Affiliations:** 1 Biological and Bio-computational Lab, Department of Life Science, Sharda School of Bio-Science & Technology, Greater Noida, Uttar Pradesh, India; 2 Department of Optometry, School of Medical and allied Sciences, Galgotias University, Greater Noida, India; 3 Department of Computer Engineering, Parul Institute of Engineering and Technology (PIET), Parul University, Vadodara, Gujarat, India; 4 School of Chinese Materia Medica, Beijing University of Chinese Medicine, Beijing, China

**Keywords:** Triple negative breast cancer, KIFC1, natural compounds, MD simulation, principal component analysis

## Abstract

TNBC is an aggressive and various subtype of breast cancer, notable by the lack of specific oestrogen receptor (ER), progesterone receptor (PR), and human epidermal growth factor receptor 2 (HER2), consequential in limited treatment options and poor prognosis. Kinesin Family Member C1 (KIFC1), a mitotic motor protein critical for centrosome clustering and spindle formation, has critical role in TNBC progress. In this situation, natural compounds were explored as probable inhibitors of this protein. we utilized molecular docking, ADMET profiling, density functional theory calculations, molecular dynamics simulations, MM/GBSA binding free energy analysis, and principal component analysis to thoroughly evaluate binding affinity, stability, and drug-likeness property of natural compounds against KIFC1. Of the 36,900 compounds utilized, five natural compounds were carefully chosen for further assessment. All five compounds Fosfocytocin, Molybdopterin Compound Z, 5-amino-2-(3-hydroxy-13-methyltetradecanamido) pentanoic acid, TMC-52A, and Muscimol exhibited significant inhibitory efficacy against KIFC1. These compounds demonstrated persistent interactions with critical residues and had advantageous binding properties in computational evaluations. The results collectively indicate their potential as effective inhibitors for targeting KIFC1 in forthcoming studies. These data collectively identify all five natural compounds as possible inhibitors of KIFC1. Nonetheless, their effectiveness and safety must be confirmed through *in vivo* and *in vitro* study prior to consideration for clinical application.

## Introduction

“Cancer has been a widespread problem around the world for centuries, and today it affects everyone from children to elderly. The fear is that if a concrete solution is not found soon, it will take the lives of countless people”.

Breast cancer (BC) classified into specific molecular subtypes according to the presence or absence of hormone receptors (ER, PR) and the HER2 protein (Effi et al., 2016; Singh et al., 2022; Singh et al., 2025). Basically, Luminal A, Luminal B, HER2^+^ and Triple negative (TN) are subtypes of BC. These tumors exhibit poor differentiation and pose significant treatment challenges, among them TNBC is the most intricate of all BC types (Ensenyat-Mendez et al., 2021; AlFayi et al., 2025). Recent available data on GLOBOCAN 2022 indicates that BC is the most prevalent cancer worldwide, with 2,296,840 new cases, and it ranks first in incidence, with an age standardized rate (ASR) of 46.8 per 100,000. It occupies the fourth position in cancer related mortality, with 666,103 fatalities and an age standardized rate of 12.7 per 100,000 individuals (https://gco.iarc.who.int/en).

TNBC noted for its aggressive and dynamic nature (Singh et al., 2024). TNBC Patients experience reduced OS (overall survival) and an increased likelihood of early recurrence, especially during the initial 3 years (Yadav et al., 2024), mostly observed in 40 years aged women, particularly with mutation in BRCA1/2, and women of Black or Hispanic descent. 19.5% of patients with TNBC had BRCA1/2 mutations (Daly et al., 2016; Singh et al., 2023). TNBC have significant treatment challenges owing to its extensive genetic diversity, heterogeneity, and propensity to acquire resistance to conventional therapy (Mahmoud et al., 2022). Presently, first line therapy comprises chemotherapeutic drugs including anthracyclines, alkylating agents, and taxanes. Recently, targeted medicines such as FDA-approved inhibitors of programmed cell death protein-1 (Keytruda), programmed death ligand-1 (Tecentriq), PARP inhibitors (Khan et al., 2025), and antibody-drug conjugates like Trodelvy have demonstrated potential in enhancing outcomes for a certain group of TNBC patients (Lau et al., 2022; Subhan et al., 2023). These drugs work by targeting key genetic changes and signaling pathways involved in tumor progression. Despite these successes, there remains a demand for more efficacious and personalized medicines, particularly for patients with recurrent or chemotherapy-resistant TNBC.

Recent studies have revealed complementary molecular targets including EGFR, VEGF, AR, ERβ, PI3K, mTOR, and AKT (Li et al., 2022). For the identification of novel target currently various methods utilizing, among them differentially expressed genes (DEGs) one of them that help in identification of new target, and clearly differentiate between healthy and patients. Data from the Gene Expression Omnibus (GEO) is being utilized with various bioinformatics tools and online web server to find DEGs in TNBC. These DEGs participate in multiple essential pathways that facilitate the development of cancer. A study done by Murtada K. Elbashir and colleagues and they identify the following essential genes: CDK1, KIF11, CCNA2, TOP2A, ASPM, AURKB, and CCNB2, that have role in BC (Elbashir et al., 2023).

Kinesins are a group of motor proteins involved in numerous functions within cellular biology, particularly in intracellular transport and cellular dynamics. Among this group KIFC1 (Kinesin Family Member C1) is notice as a pivotal protein in cancer biology. KIFC1 is crucial for the assembly of the mitotic spindle, chromosomal segregation, and cellular migration. The anomalous expression of KIFC1 correlates with tumour cell proliferation, metastasis, and adverse clinical outcomes across various cancer. This protein mechanistically influences critical signalling pathways related to cell cycle control and apoptosis (Sanghvi et al., 2025). its located-on chromosome 6p21.32. KIFC1 employs ATP hydrolysis to enable the transport of vesicles and organelles along microtubules, basically KIFC1 consists of 663 amino acids and is categorized into three primary regions a tail domain (residues 1-138), a coiled-coil region (residues 141-297), and a motor or head domain (residues 317-663) (She and Yang, 2017). Biophysical and Structural study specify that KIFC1 operates as a dimer, characterized by an α-helical coiled-coil stalk that links a structured motor domain to a flexible tail region. Dimerization of coiled-coil domain specifically affects KIFC1’s kinetic properties. Kinesins have two specific inhibitor-binding sites α4/α6 cleft and L5/α2/α3 pocket. Compounds that target this region often function via allosteric, ATP-non-competitive processes, modifying ADP release without directly affecting ATP binding (Sharma et al., 2023). KIFC1 have role in spindle formation during mitosis by sliding and crosslinking microtubules, hence maintaining accurate spindle architecture. Additionally KIFC1 have role in the cargo transport towards the minus end of microtubule and also has the capacity to bind dsDNA, that indicating its role in transporting foreign DNA into the nucleus (Farina et al., 2013). Its notably significant in the realm of malignancy. Tumor cells use this ability to transform multipolar spindles into pseudo-bipolar spindles, enabling their survival during mitotic aberration. These adjustments of the spindle facilitate accurate chromosomal segregation and diminishes the likelihood of mitotic errors that may result in cellular apoptosis. It was notice that KIFC1 is non-essential in normal cells but is crucial for the survival of tumor cells with amplified centrosomes, rendering it an attractive and selective target for cancer treatments (Sanghvi et al., 2025).

Numerous inhibitors developed for kinesin, including SR31527, CW069, and AZ82; however, their limited strength and selectivity have hampered their clinical progress (Fan et al., 2021). In the treatments such as chemotherapy and ionizing radiation can paradoxically activate the ATM/ATR-KIFC1 phosphorylation pathway, thus facilitating centrosome clustering and increasing cancer cell survival, that contributes to malignancy recurrence and growth, metastasis (Fan et al., 2021; Sharma et al., 2023). various preclinical studies suggest that AZ82 specifically inhibits tumor development *in vivo* with minimal toxicity and promoting senescence through the upregulation of two special protein p53 and p21, simultaneously downregulating cyclin D1, p-Rb, and CDK4 (Lu et al., 2024). SR31527 inhibits spindle formation in cancer cells, while PJ34 suppresses KIFC1 expression, thereby reducing cell proliferation. Similarly, other drugs like DOX in combination with abemaciclib rapidly decreases tumor growth. Despite these efforts, nowadays no promising candidate has been identified that can successfully target and inhibit and manage this disease. Recent studies results indicate that natural product (NP) have potential to significantly target cancer related proteins and can manage TNBC (Zhang et al., 2016).

NP have been used for centuries to address a variety of health disease. Compounds derived from natural source like plants, fungi, microbes and sea animals have historically been valued for their medicinal properties (Chopra and Dhingra, 2021). NP exhibit potent anti-cancer effects, including the ability to reduce cancer aggressiveness, inhibit the proliferation and metastasis in malignant cells, and also regulate cancer related pathways (Mitra and Dash, 2018). NP product curcumin, diosgenin, garcinol, genistein, honokiol, quercetin, resveratrol, silibinin, tetrandrine, and thymoquinone noted for the disease management (Yang et al., 2022; Kataria et al., 2024). NP play multiple mechanisms, like the promote apoptosis, inhibition of angiogenesis, induction of cell cycle arrest, and disruption of specifics signaling pathways, that rendering them significant candidates in the advancement of more effective and safer BC therapies (Noel et al., 2020). These characteristics provide NP may interesting candidates for management KIFC1.

The present work is original study focused on identification of a natural compounds that can potentially inhibit KIFC1 protein a potential target of TNBC.

## Materials and methods

### Target protein optimization

KIFC1, related to the kinesin-14 family, and plays specific crucial role in spindle assembly and chromosome segregation during mitosis. Crystal structure (PDB ID: 5WDH) was elucidated by X-ray diffraction at a resolution of 2.25 Å. ADP occupies the nucleotide-binding site as the protein’s natural ligand. The binding of ADP offers valuable insights into the conformational dynamics of KIFC1 and its molecular interactions with nucleotides.

Co-crystal structure was prepared using the Protein Preparation Wizard in Schrödinger’s Maestro suite, applying default parameters. Pre-processing included the addition of hydrogen atoms, removal of unnecessary hydrogens, and assignment of zero-order bonds to metals and disulphide bonds. H2O molecules located more than 5.0 Å from any hetero group were deleted, Protonation states were predicted using Epik at a target pH of 7.0 ± 2.0. Additionally, no heteroatom groups were retained to ensure a pristine structure for docking. In final refinement, H2O orientations were sampled and hydrogen bonds optimized utilizing PROPKA (pH 7.0), followed by restrained minimization of heavy atoms to an RMSD of 0.30 Å with the OPLS4 force field. Resulting energy-minimized protein structure was subsequently used for molecular docking (Madhavi Sastry et al., 2013). After the optimization of the target protein for stability and precision, a library of NP was prepared to find potential candidate that exhibiting favorable biological activity and target KIFC1.

### Natural compounds library

NP are retrieved from Natural Products Atlas (https://www.npatlas.org/), that is a curated open-access database of NP, these compounds were processed with the LigPrep module inside the Schrödinger Suite, employing the OPLS4 force field (Yusuf et al., 2025) for the structural optimization. Additionally, Epik was utilized to produce potential stereoisomers, ionization states, and tautomeric forms at a physiological pH of 7.0 ± 2.0. Moreover, a maximum of five low-energy ring conformations were produced for each candidate to guarantee structural variety for ensuing docking analyses (Yusuf et al., 2025).

### Active site determination

In *in silico* approach structure-based drug design focuse on specific binding sites on target proteins to facilitate the development of compounds that inhibit their biological function, resulting disrupting cellular processes (Klebe, 2000). To KIFC1, the active site was delineated by analyzing the interactions between co-crystallized ligands and key amino acid residues. In the detailed interaction profiling revealed several critical residues that stabilize the ligand through hydrogen bonding and hydrophobic interaction. Notably, hydrogen bonds were observed between ADP and five critical residues like Gly413, Gly415, Lys416, Thr417, and Phe418 establish hydrogen bonds predominantly with the oxygen atoms of the ADP molecule, that suggesting robust and unique interactions that probably enhance the stability and binding affinity of ADP in the active site. Moreover Arg316, Arg318, Pro319, Leu321, Thr412, and Ser414 engage with ADP via hydrophobic interactions and electrostatic stabilization, facilitating its binding at the active site.

Receptor grid was generated utilizing Glide module in Maestro with default parameters. The grid was centered on the active site residues identified from ligand interaction analysis. The software automatically estimated the grid box dimensions based on the spatial distribution of the residues. The additional parameters, including van der Waals scaling (1.0), partial charge cutoff (0.25), and input partial charges, were maintained at their default settings. After these settings structure-based virtual screening was conducted utilizing the defined active site to assess the binding affinity of the NP’s library.

### Structure-based virtual screening (SBVS)

Structure-based virtual screening was done in three consecutive phases: first High-Throughput Virtual Screening (HTVS), second Standard Precision (SP), and last Extra Precision (XP) docking. Primely, HTVS was utilized to rapidly filter out the majority of NP, refining the dataset by picking only the top 10% of candidates for the subsequent SP and XP docking phases. These systematic screening methods helped the effective identification of novel potential ligands, increasing accuracy at each level, and this hierarchical methodology has demonstrated efficacy in targeting the KIFC1 protein by balancing computational efficiency with the dependability of binding predictions (Hassan et al., 2023). After that selected top compounds from the virtual screening consider for additional analysis like their physicochemical and pharmacokinetic propertese via drug-likeness assessment.

### Drug-likeness evaluation

Medicinal properties and drug-likeness of the screened NP were evaluated via ADME studies utilizing the SwissADME web tools (http://www.swissadme.ch/). This valuation provides essential insights into the compound’s potential for advanced medication development and refinement. SwissADME evaluates essential medicinal properties, like water solubility, molecular kinetics, lipophilicity, and drug likeness (Daina et al., 2017). Additionally, it also evaluates adherence to Lipinski’s rule of five, that encompasses crucial parameters including a molecular weight under 500, a QPlogPo/w below 5, fewer than 5 hydrogen bond donors, and 10 hydrogen bond acceptors, moreover, toxicity prediction was conducted concurrently to evaluate the safety profile of the selected NP.

### Toxicity prediction

Online web server tool ProTox 3.0 utilized to assess the probable toxicity profiles of the leads NP (https://tox.charite.de/protox3/). This tool publicly available web server for toxicity prediction that combines molecular similarity analysis with machine learning models to evaluate many toxicity endpoints (Banerjee et al., 2024; Kumar et al., 2024b). ProTox 3.0 accessible extensive toxicity predictions across sixty-one endpoints, like acute toxicity (LD_50_), organ toxicity, carcinogenicity, cytotoxicity, mutagenicity, and immunotoxicity. These step was crucial for identifying and characterizing potentially toxic molecules, ensuring that only compounds with beneficial toxicity profiles were retained for further investigation (Banerjee et al., 2024).

After virtual screening and docking, those compounds satisfied the specified inclusion criteria like molecular weight less than 500 Da, hydrogen bonds donors less than 5 and acceptor bonds 10, LogP value less than 5, and rotatable bonds are 10 they successfully underwent ADME based filtering criteria. Additionally, those ligands exhibiting greater docking scores in compression to the control and analogous amino acid interactions in both 2D and 3D analyses were chosen. Moreover, the superimposition with the co-crystallized ligand confirmed that these compounds occupied the identical binding pocket with an RMSD under 2.0 Å, and they were subsequently employed for DFT and molecular dynamics simulations to determine stability and binding reliability.

### Density functional theory (DFT)

Density Functional Theory were utilized to assess the electrostatic characteristics of the leading NP. The screened NP structures were loaded into the Maestro module and evaluated with Jaguar software (Bochevarov et al., 2013). Highly stable conformation of each molecule was initially determined using the Macromodel function in Jaguar. Optimized structures were analyzed by single-point energy calculations employing the B3LYP/6-31G* hybrid functional. This method eased the identification of molecular orbital surfaces and electron density distributions (Kumar et al., 2024a).

### Molecular dynamics (MD) simulation

Molecular dynamics simulations were performed using the Desmond module, that evaluate the stability and interaction strength of the protein-ligand docked complexes. Each simulation was conducted for a 100 ns (Bowers et al., 2006; Kumar et al., 2022). These docked complexes were solubilized in an orthorhombic box containing TIP3P water molecules, with dimensions of 10 × 10 × 10 Å^3^. The systems were neutralized with Na^+^ or Cl^−^ counter-ions at a concentration of 0.15 mol/L to simulate physiological environments (Kumar et al., 2024b; Kumar Tiwari et al., 2024). Additionally, Energy minimization was done by Steepest Descent and Conjugate Gradient methods. Primirly, constraints were imposed on the solute, and the system was reduced for up to 2000 steps using a convergence criterion of 1.0 kcal/mol/Å (Kaczor et al., 2015). Following minimization was executed utilizing the Broyden-Fletcher-Goldfarb-Shanno (LBFGS) algorithm, and minimized systems were subsequently equilibrated at a temperature of 300 K and a pressure of 1.013 bar, with relaxation durations of 1 ps and 2 ps, respectively. The Berendsen NVT and NPT ensembles were utilized for the equilibration of temperature and pressure. During the simulation, a constant temperature and pressure were maintained using the Nose-Hoover thermostat and the Martyna-Tobias-Klein barostat. RESPA integrator was utilized for the simulation with a 2 femtosecond (2-fs) time step, additionally long-range electrostatic interactions were calculated utilizing the Particle Mesh Ewald (PME) technique (Shukla et al., 2023). Following to the simulation trajectories were analyzed for various molecular dynamics parameters, like root mean square deviation (RMSD), root mean square fluctuation (RMSF), and protein-ligand interactions. These evaluations were directed by the using Simulation Interaction Diagram (SID) module (Harder et al., 2016).

### Calculations of binding free energy

Prime MM/GBSA method was utilized for the calculations of binding free energy that is module of Schrödinger suite, along with VSGB 2.0 solvation model, for the calculation of the binding free energy of the top ligand-protein complexes. For this we used 200 frames from the last 20 ns of the simulation to look at the ΔG-binding energy of each protein-ligand combination. As part of the MM/GBSA investigation, additionally we estimated a number of factors, like hydrogen bonding, Coulombic interactions, lipophilicity, van der Waals contacts, and generalized Born electrostatic solvation (Gupta et al., 2022). MM/GBSA were used for the analysis to find the average binding free energy values and then looked at the standard deviation to see how reliable the energy estimations were. Thermal mmgbsa.py tool from the Schrödinger suite was used to find the net binding free energy for each selected complex.

$$\text{MM}/\text{GBSA}\;\Delta G_{\text{bind}}=\Delta G_{\text{complex}}-\Delta G_{\text{protein}}-\Delta G_{\text{ligand}}$$

Symbols like ΔGcomplex, ΔGprotein, and ΔGligand symbolize the free energies of the ligand-protein complex, the unbound protein, and the unbound ligand, respectively. A less (in negative) ΔGbind value suggests a more robust and advantageous binding affinity of the compounds for the target protein.

Principal component analysis was carried out on the molecular dynamics trajectories to explore the reliable motions and structural changes taking place within the complexes.

### Principal component analysis (PCA) of MD simulations

PCA was conducted to examine the essential dynamics of each complex derived from molecular dynamics (MD) simulations. MD simulations were carried out using the Flare module of Cresset, that were utilized for the standardized system preparation and default simulation protocols to ensure reproducibility. Primary objective was to explore the conformational landscape and identify the central motions leading complex interactions (Niharika et al., 2024). After the completion of the simulations, trajectory data were subjected to PCA using Flare’s integrated analytical tools. PCA can simplify complex motion data by breaking down the atomic displacement covariance matrix into a set of independent eigenvectors, or principal components. Each component has an eigenvalue that reflects how much movement occurs in that specific direction. The first few components, which account for the largest variations in atomic positions, were then examined to identify the key conformational changes that took place during the simulation period. This analysis enabled the identification of collective motions and potential allosteric shifts critical for ligand recognition and binding. The reduced dimensionality representation of the trajectory provided insights into functional mechanisms and the dynamic behavior of the complex. Additionally, the analysis of correlated and anti-correlated atomic motions derived from the covariance matrix revealed inter residue communication patterns that may be essential for biological activity. All procedures, including simulation execution, trajectory processing, and PCA, were performed within the Flare environment to maintain analytical consistency and ensure data integrity throughout the workflow.

**FIGURE 1:**
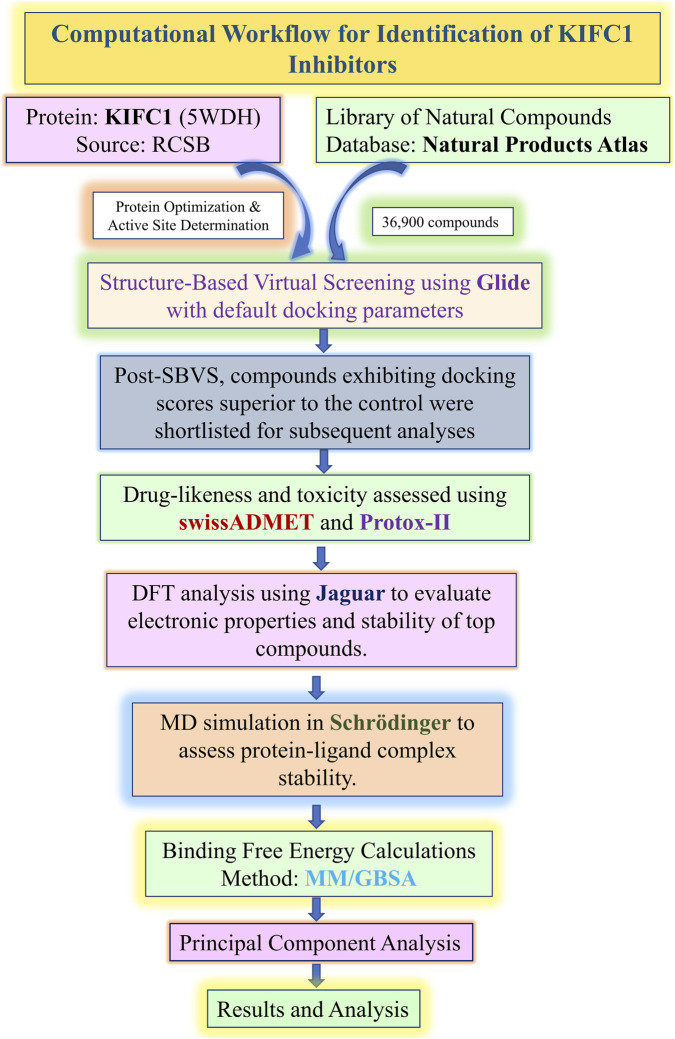
Computational workflow for identification of KIFC1 inhibitors.

A schematic Diagram of the computational method which employed in this study is presented in Figure 1.

## Results

Principal aim of this study is to inhibit the function of the KIFC1 protein, for this NP were chosen for potential inhibitors of the KIFC1 protein, NP have structural diversity, high biocompatibility, and minimal toxicity compared to synthetic molecule (Barnes et al., 2016). It was notice that, they have evolved over time to effectively interact with biological systems of animals including human, these characteristics making them an attractive candidates for medicinal discovery (Barba-Ostria et al., 2022; Ding and Xue, 2024). In this study NP obtained from the Natural Products Atlas database and virtual screening was performed against the KIFC1 protein using the Glide module of the Schrödinger suite, as detailed in the methodology.

For the control docking co-crystallized ligand (ADP) of the KIFC1 protein were utilized that confirm the molecular docking methodology. Reference compounds were re-docked into the protein’s active site utilizing Glide (Schrödinger Maestro) with default parameter resulting docked pose successfully replicated the critical amino acid interaction initially identify in the crystal structure. These encompass essential residues, including Gly413, Gly415, Lys416, Thr417, and Phe418, which are involved in hydrogen bonding. In addition, Arg316, Arg318, Pro319, Leu321, and Thr412 facilitate ADP binding via hydrophobic and electrostatic interactions, substantial resemblance between the docked and crystallographic interactions confirmed the reliability and precision of the docking configuration. Additionally, structural superimposition of the docked and crystal conformations demonstrated a noteworthy degree of overlap, thereby validating the precision of the docking process (Supplementary Figure S1). This method was subsequently utilized for the virtual screening of a NP to investigate potential inhibitors of KIFC1.

Among the screened NP, 12 showed strong binding interactions with the KIFC1 protein. These compounds demonstrated docking scores ranging from −7.01 to −7.86 and glide energy 302 between −24.67 and −60.91 kcal/mol. It showed more strength than the reference ligand (docking score: -6.78 kcal/mol; glide energy: -51.14 kcal/mol), (Supplementary Table S1). Remarkably, TMC-52A showed the most promising binding affinity with a docking score of −7.86, and a Glide Energy of 60.91. Fosfocytocin followed closely, with a docking score of −7.85 and glide energy −57.94. Similarly, 5-amino-2-(3-hydroxy-13-methyltetradecanamido) pentanoic acid exhibited a docking score of −7.687, with a Glide Energy of −53.292 indicating moderate binding potential. Additionally, Molybdopterin Compound Z and Muscimol showed docking scores of −7.38 and −7.25, respectively, with corresponding Glide energy of −45.51 and −24.67 (Table 1). For the validation of these top compounds bind at the same active site as the reference compound, superimposition studies were done on the top five docked ligands, and results confirmed that the identified NP were consistently aligned with the binding site of the reference compounds and interacted with almost similar amino acid residues (Supplementary Figure S2). For the further strengthen these resluts the inhibitory potential of the top candidates against the KIFC1 protein, additional validation was conducted using multiple computational tools.

**TABLE 1:** Top 5 compounds with their docking score, Glide GScore, and interacting amino acids.

ID	Compound_id	Compound_name	Docking score	Glide energy	Interacting amino acid
NPA009447	9447	TMC-52A	−7.862	−60.913	gln411, thr412, gly413, ser414, gly415, lys416, thr417, phe418, glu421, gly423, pro424, arg318, leu321, arg524, arg528, tyr461, ser535, ser536, ser538, ser540, asp565, leu566, ala567
NPA021320	21,320	Fosfocytocin	−7.857	−57.934	gln411, thr412, gly413, ser414, gly415, lys416, thr417, phe418, arg528, ser535, ser536, arg318, pro319, leu321, pro424
NPA034881	34,881	5-amino-2-(3-hydroxy-13-methyltetradecanamido) pentanoic acid	−7.687	−53.292	gln411, thr412, gly413, ser414, gly415, lys416, thr417, phe418, asp565, leu566, ala567, ser535, ser536, ser538, ser540, pro424, gly423, gln384, gly383, ser382, gly381, pro380, arg316, arg318, pro319, leu321, leu430
NPA016275	16,275	Molybdopterin compound Z	−7.384	−45.513	gln411, thr412, gly413, ser414, gly415, lys416, thr417, phe418, leu321, arg318, pro424, gly423, leu430, ser536
NPA024780	24,780	Muscimol	−7.25	−24.679	thr412, gly413, ser414, lys416, thr417, ser535, ser536, ser538, ser540, asp565, leu566, ala567
PubChem CID	6022	Adenosine 5′-diphosphate	−6.780	−51.143	gln411, thr412, gly413, ser414, gly415, lys416, thr417, phe418, ser536, arg318, leu321, pro424, gly423, leu430

### ADME profile of compounds

Compounds designated for medical application must have drug-like characteristics and advantageous pharmacological attributes, such as solubility, permeability, metabolic stability, and interactions with transport proteins. The SwissADME web tool was utilized to assess the Absorption, Distribution, Metabolism, and Excretion (ADME) properties and drug-likeness of the selected compounds. This investigation yielded information regarding their pharmacokinetic characteristics. The ADME characteristics of the five leading compounds TMC-52A, Fosfocytocin, 5-amino-2-(3-hydroxy-13-methyltetradecanamido) pentanoic acid, Molybdopterin Compound Z.

The ADME investigation indicated that all five compounds were non-toxic and non-carcinogenic to human cells. Cytochrome P450 2D6 (CYP2D6), which metabolizes roughly 20%–25% of pharmaceuticals and xenobiotics in the human liver, may result in increased drug concentrations in the bloodstream when blocked. Nonetheless, none of the chosen drugs were anticipated to inhibit CYP2D6 or other cytochrome enzymes, as evidenced by SwissADME data.

NP exhibit unique medicinal properties like TMC-52A and Fosfocytocin demonstrate gastrointestinal absorption and lack blood-brain barrier permeability. On the other hand, the amino acid derivative exhibits significant lipophilicity, elevated gastrointestinal absorption, absence of RO5 violations, and modest synthetic viability. Molybdopterin and Compound Z shows good solubility, gastrointestinal absorption. Muscimol is smallest and most accessible compound that have high gastrointestinal absorption, excellent solubility, and full adherence to the Rule of Five, however it is deficient in blood-brain barrier permeability (Supplementary Table S2). Selected compounds show significant variability in drug-likeness, solubility, and absorption capacity, that underscoring their broad therapeutic potential (Table 2).

**TABLE 2:** ADME attributes of natural compounds.

Properties		TMC-52A	Fosfocytocin	5-amino-2-(3-hydroxy-13-methyltetradecanamido) pentanoic acid	Molybdopterin compound Z	Muscimol
	Formula	C20H30N4O6	C12H20N4O13P2	C20H40N2O4	C10H10N5O7P	C4H6N2O2
Physicochemical properties’	MW (g/mol)	422.48	490.25	372.54	343.19	114.10
	Heavy atoms	31	37	27	25	8
	Rotatable bonds	16	11	18	2	1
	H-bonds acceptors	8	14	5	10	3
	H-bonds donor	6	6	4	4	2
Lipophilicity	C Log Po/w	−0.3	−3.45	2.89	−1.83	−0.54
Water solubility	ESOL Log S	0.17	0.83	−2.11	−0.39	0.56
Pharmacokinetics	GI absorption	Low	Low	High	Low	High
	BBB permeant	No	No	No	No	No
Drug-likeness	RO5 violation	1	2	0	1	0
Medicinal chemistry	Synthetic accessibility	4.17	5.21	3.93	3.92	2.23

After ADME profiling, the toxicity profiles of the compounds were further exploring to determine their overall safety.

### Toxicity profile of compounds

Following to ADME profiling, the toxicity potential of the compounds was further investigated utilising the ProTox 3.0 online server. TMC-52A demonstrated oral LD_50_ values 2100 mg/kg while rest compounds LD_50_ values were 1190, categorising them into toxicity classes 5 and 4, which signify practically non-toxic characteristics. Fosfocytocin, 5-amino-2-(3-hydroxy-13-methyltetradecanamido) pentanoic acid, Molybdopterin compound Z, and Muscimol exhibited slightly hepatotoxicity and immunotoxicity with moderate value. TMC-52A demonstrated modest mutagenicity and clinical toxicity (probabilities ∼0.55-0.64), although these figures are below acceptable safety thresholds for early-phase screening (Supplementary Table S3). All five compounds were consistently predicted to be non-carcinogenic, non-cytotoxic, and inactive for important CYP450 metabolic enzymes, hence affirming their favorable safety profiles and appropriateness for further pharmacological research (Table 3). Following the evaluation of toxicity profiles, these compounds selected for subsequent docking studies to investigate their binding affinity and interaction patterns with the target protein.

**TABLE 3:** Toxicity profiling of natural compounds.

Compound	Hepatotoxicity	Carcinogenicity	Immunotoxicity	Mutagenicity	Cytotoxicity	LD_50_ (mg/kg)/class
TMC-52A	Inactive (0.86)	Inactive (0.63)	Inactive (0.95)	Active (0.55)	Inactive (0.70)	2100 (5)
Fosfocytocin	Active (0.69)	Inactive (0.62)	Active (0.96)	Inactive (0.97)	Inactive (0.93)	1190 (4)
5-amino-2-(3-hydroxy-13-methyltetradecanamido)pentanoic acid	Active (0.69)	Inactive (0.62)	Active (0.96)	Inactive (0.97)	Inactive (0.93)	1190 (4)
Molybdopterin compound Z	Active (0.69)	Inactive (0.62)	Active (0.96)	Inactive (0.97)	Inactive (0.93)	1190 (4)
Muscimol	Active (0.69)	Inactive (0.62)	Active (0.96)	Inactive (0.97)	Inactive (0.93)	1190 (4)

### Docking analysis of TMC-52A

**FIGURE 2:**
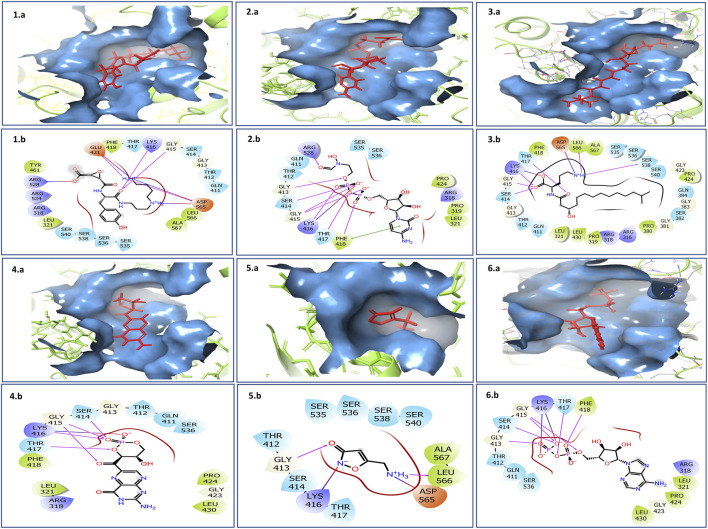
Three- and two-dimensional docked complexes of the selected natural compounds, specifically (1. a, 1. b) TMC-52A, (2. a, 2. b) Fosfocytocin, (3. a, 3. b) 5-amino-2-(3-hydroxy-13-methyltetradecanamido) pentanoic acid, (4. a, 4. b) Molybdopterin compound Z, (5. a, 5. b) Muscimol, and (6. a, 6. b) reference complex Adenosine 5′-diphosphate, exhibit binding at the active site of the KIFC1 protein. The two-dimensional representations document essential interactions, including hydrogen bonds (pink arrows), hydrophobic interactions (green), polar residues (blue), negatively charged residues (red), glycine (grey), and salt bridges (red and blue) for the docked complexes of KIFC1 with the chosen natural compounds.

The 2D interaction diagram of TMC-52A with its target protein KIFC1 illustrates a clearly delineated and varied network of stabilizing contacts. Ligand TMC-52A stabilized several hydrogen bonds with polar and charged amino acids, like Glu421, Asp565, Lys416, and Gly415. The interactions cover the ligand’s amine and carbonyl groups, which significantly enhance its binding affinity. A salt bridge was noted between the ligand’s carboxyl group and Arg528, supplementary enhancing the electrostatic stability of the complex. Additionally, pi-cation interactions transpire between the positively amino acid residues ring system of the ligand and the aromatic side chain of Phe418, enhancing the binding selectivity. Hydrophobic residues including Leu321, Leu566, Ala567, and Tyr461 encircle the ligand, stabilizing its shape via van der Waals interactions. Glycine amino acid residues like Gly413, Gly415 enhance backbone flexibility, enabling optimal orientation of nearby polar residues including Ser414 and Gln411, which collectively establish a polar environment that is favorable for ligand binding (Figure 2 1.a, 1.b). Compare with reference compound and TMC-52A it was notice that they shared almost similar amino acid, including Lys416, Thr417, Phe418, Gly415, Gly413, Thr412, Gln411, and Ser414. The residues are mostly engaged in hydrogen bonding and electrostatic interactions inside the binding site, which suggesting that TMC-52A occupies a comparable active site region as reference compounds. These interactions indicate that TMC-52A is securely and specifically positioned within the protein’s active region, emulating essential characteristics of nucleotide ligands and exhibiting significant promise as a lead compounds in drug development for the target of KIFC1.

### Docking analysis of fosfocytocin

Fosfocytocin establishes number hydrogen bonds with amino acid residues including Ser 414, Gly 413, Gly 415, and Lys416, which are important for specificity and high affinity. Moreover, it was also notice that other interaction like ionic interaction, or salt bridges also stabilized, transpire between positively charged residues such as Arg 318 and Arg 528 and negatively charged groups on the ligand, so enhancing the stability of the complex. Additionally, a pi-cation contact exists between the aromatic residue Phe418 and a positively charged group on the compound, that facilitating correct orientation within the binding pocket. Moreover, hydrophobic residues such as Phe facilitate nonpolar interactions that stabilize the ligand’s location. These interplay bonds like hydrogen bonds, ionic contacts, pi-cation stacking, provides a highly specialized and stable binding environment crucial for development of effective inhibitors (Figure 2 2.a, 2.b) In compression of reference compounds, almost all residues, including Ser414, Gly413, Gly415, Lys416, Arg318, Arg528, Phe418, Leu321, Pro424, and Gly423, were present.

### Docking analysis of 5-amino-2-(3-hydroxy-13-methyltetradecanamido)pentanoic acid

The 5-amino-2-(3-hydroxy-13-methyltetradecanamido) pentanoic acid has a lactone ring, an amide bond, an extensive aliphatic side chain, a hydroxyl group, and a protonated amine group (N^+^H_3_). Chemical groups engage in a sequence of hydrogen bonding. Hydrogen bond interactions are noted between the oxygen atoms of the lactone ring and the residues including Ser414, Gly415, Lys416, and Thr417. Carbonyl oxygen of the amide group creates hydrogen bond with the negatively charged residue Asp565. Amine group engages with Leu566, while a hydrogen bond is observed between an oxygen atom from the ligand’s backbone and Ser538. These interactions have role in stabilization of the ligand at the protein’s active site.

Binding pocket is enclosed by number of residues, polar residues like Ser535, Ser536, Ser538, Ser540, Thr412, and Gln411 surround the ligand and presumably facilitate ligand recognition via hydrogen bonding or dipole interactions. Charged residues, such as Lys416 and Arg316/Arg318 (positively charged) and Asp565 (negatively charged), have role in electrostatic stabilization. Hydrophobic residues such as Leu321, Leu430, Leu566, Ala567, Pro319, Phe418, Pro424, and Pro380 establish van der Waals or hydrophobic interactions with the ligand’s, therefore securing it within the binding site. Glycine residues like Gly413, Gly415, Gly381, Gly383, and Gly423, recognized for their structural flexibility, and facilitate the accommodation of the ligand’s conformation within the binding pocket (Figure 2 3.a, 3.b). In compression of reference compounds, almost all residues are present.

### Docking analysis of molybdopterin

Molybdopterin held within the binding pocket via hydrogen bonds, electrostatic interactions, and hydrophobic contacts. Crucial hydrogen bonds are established between the phosphate and ribose oxygen atoms of the compounds and the residues like Ser414, Gly415, Thr417, and the positively charged such as Lys416. Lys416 engages in a pi-cation interaction with the ligand’s aromatic ring structure, contributing further electrostatic stability. Adjacent polar residues, including Gln411 and Ser536, facilitate further polar interactions, but the flexible glycine residues Gly413 and Gly415 enable tight packing around the ligand. Hydrophobic residues such as Phe418, Leu321, Leu430, Pro424, and Pro319 establish a nonpolar milieu that enhances the ligand’s hydrophobic surfaces (Figure 2 4.a, 4.b). In compression of reference compounds, molybdopterin exhibit interactions with almost all the same crucial amino acid residues including Lys416, Thr417, Ser414, and Gly415 via hydrogen bonding and electrostatic interactions. However, the ligands establish more comprehensive interactions, encompassing pi-cation contacts and engagement with residues such as Asp565 and Leu566, indicating a potentially stronger or more stable binding inside the active site of protein.

### Docking analysis of muscimol

Muscimol, a small compound, that engages via hydrogen bonds and pi-cation interactions, thereby cementing its position within the binding pocket of protein. Critical amino acid includes Lys16, which establishes a robust pi-cation contact with the aromatic component of muscimol, and Leu566, which forms a hydrogen bond with the protonated amine group of the ligand. Supplementary hydrogen bonds are detected with Gly413 and Ser414, reinforcing the ligand’s anchorage. Negatively charged residues like Asp565 is nearby, possibly aiding in electrostatic stability. Residues like Ser535 and 540, Ala567, and Thr412 and 417 create the adjacent polar environment, enabling ligand compatibility (Figure 2 5.a, 5.b). Interaction profile indicates a persistent binding orientation, but less widespread than that of bigger ligands with many binding domains. Compared to the reference compounds, Muscimol engages with multiple identical key residues that was present in reference compounds specifically Lys416, Gly413, Ser414, Asp565, and Leu566.

### Docking analysis of reference compounds

Reference compounds has a complex interaction profile within the protein’s active site region that establishing several hydrogen bonds and ionic interactions mainly via its phosphate and sugar moieties. Critical residues occupied are Gln411, Thr412, Gly413, Ser414, Gly415, Lys416, Thr417, and Phe418, underscoring significant polar and ionic interactions. Additionally, Hydrophobic residues, including Arg318, Leu321, Pro424, and Leu430, contribute to further stabilization (Figure 2 6.a, 6.b).

### Density functional theory (DFT) analyses of leading compounds

DFT analysis was carried out to investigate the electronic properties of the key compounds TMC-52A, Fosfocytocin, 5-amino-2-(3-hydroxy-13-methyltetradecanoamido) pentanoic acid, molybdopterin compound Z and muscimol. Energies of the HOMO (Highest Occupied Molecular Orbital) and LUMO (Lowest Unoccupied Molecular Orbital) were computed to assess their charge transfer capability, chemical reactivity, and stability. The energy gap (ΔE = LUMO - HOMO) indicates the electronic behaviour of the molecule.

**FIGURE 3:**
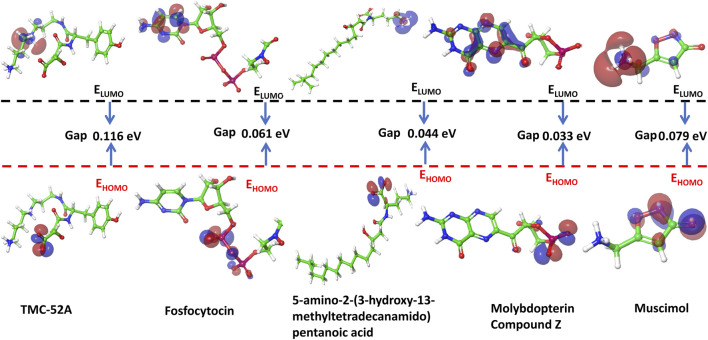
Frontier molecular orbital (HOMO-LUMO) and related energy of top 5 compounds such as TMC-52A, Fosfocytocin, 5-amino-2-(3-hydroxy-13-methyltetradecanamido) pentanoic acid, Molybdopterin compound Z, Muscimol and reference compound - Adenosine 5′-diphosphate.

TMC-52A demonstrated the lowest HOMO (−0.244572 eV) and LUMO (−0.128282 eV) values, yielding the greatest energy gap of 0.116290 eV, which signifies robust kinetic stability and decreased reactivity. Fosfocytocin showed HOMO and LUMO energies of −0.113532 eV and −0.052530 eV, giving an energy gap of 0.061002 eV, indicating moderate reactivity. The compound 5-amino-2-(3-hydroxy-13-methyltetradecanamidho) pentanoic acid showed a HOMO value of −0.123920 eV and LUMO value of −0.079149 eV, giving an energy gap of 0.044771 eV, indicating a moderately high electron-donating ability. Molybdopterin compound Z exhibited the smallest energy gap, 0.033128 eV, derived from its HOMO (−0.036697 eV) and LUMO (−0.003569 eV) energies, marking it as the most chemically active compound in the group. Muscimol recorded HOMO and LUMO energies of −0.133248 eV and −0.054146 eV, respectively, with an energy gap of 0.079102 eV, suggesting moderate reactivity. Among the analyzed molecules, TMC-52A was found to be the most stable, while molybdopterin compound Z showed the strongest chemical reactivity (Figure 3) (Table 4).

**TABLE 4:** A comparative analysis of the HOMO and LUMO orbital energy levels of the selected compounds.

Compound	HOMO (eV)	LUMO (eV)	Energy gap (eV)
TMC-52A	−0.244572	−0.128282	0.116290
Fosfocytocin	−0.113532	−0.052530	0.061002
5-amino-2-(3-hydroxy-13-methyltetradecanamido) pentanoic acid	−0.123920	−0.079149	0.044771
Molybdopterin compound Z	−0.036697	−0.003569	0.033128
Muscimol	−0.133248	−0.054146	0.079102

After the analysis of the DFT results, further molecular dynamics (MD) simulations were conducted to assess the dynamic stability and conformational behavior of the protein-ligand complex under physiological parameters.

### Molecular dynamics simulation data analysis

Molecular dynamics simulation was conducted to evaluate the time dependent stability of the complexes, which providing insights into the protein’s dynamic characteristics and structural alterations during ligand binding. Over a 100 ns simulation period, several parameters including RMSD, RMSF and protein-ligand interaction patterns were analyzed to evaluate the overall stability of the complexes. While RMSD (Root Mean Square Deviation) help the observation of global structural alterations, emphasizing comprehensive conformational changes within the docked complexes. On the other hand, RMSF (Root Mean Square Fluctuation) emphasizes local movements by monitoring the atomic level motion of the ligand and each protein amino acid residue during their interaction. Additionally. The binding stability of ligands at the active site was assessed via protein-ligand contact analysis, which quantifies enduring connections during the simulation period. Moreover, 3D surface analysis of the final structure after 100 ns simulation revealed significant structural changes in KIFC1 when bound to natural compounds, compared to its interaction with the reference molecule ADP. These observations suggest that natural compounds have the ability to substantially modify the native structure of the KIFC1 protein.

### RMSD and RMSF analyses

**FIGURE 4:**
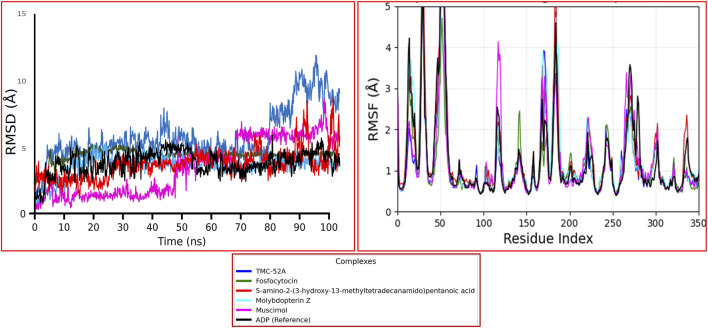
RMSD and RMSF plots of the KIFC1 protein in complex with the selected natural compounds over the 100-ns molecular dynamics simulation. The color scheme represents: blue (TMC-52A), green (Fosfocytocin), red (5-amino-2-(3-hydroxy-13-methyltetradecanamido) pentanoic acid), cyan (Molybdopterin Z), magenta (Muscimol), and black (ADP, reference). The left panel shows the backbone RMSD, and the right panel shows residue-wise RMSF for each complex computed from the same 100-ns trajectory.

The RMSD of the complexes were evaluate over 100 ns to determine structural stability (Figure 4). The reference complex (black line) showed stability during the simulation, this way, the reliability of the simulation approach is confirmed and a reference point for comparison is presented. Among the study ligands, like TMC-52A (blue) and 5-amino-2-(3-hydroxy-13-methyltetradecanamido) pentanoic acid (red) demonstrated stable trajectories after approximately 15 ns, sustaining average RMSD values between 2.0 and 2.5 Å, that signifying well-converged binding and minimal deviation from the initial conformations. Molybdopterin Z (cyan) and muscimol (magenta) showed moderate fluctuations of about 3–3.5 Å and got stability after about 40 ns, suggesting minor rearrangements in the loop regions near the active site before equilibrium. In contrast, Fosfocytocin (green) displayed higher RMSD values (∼5 Å), indicating more pronounced structural changes, likely caused by reorientation of flexible residues around the binding pocket of protein. All complexes reached equilibrium within the simulation period, however the degree of deviation differed that depending on each ligand’s size and interaction pattern. Resulting consistent RMSD pattern observed for TMC-52A and the reference complex suggests that these ligands maintain the protein’s structural framework with minimal disturbance, consistent with the conformational adaptability reported in similar enzyme systems.

Further the understanding of residue-level flexibility and identify regions contributing to these conformational shifts, RMSF analysis was utilized for selected protein ligands complexes.

The RMSF profiles of the protein-ligand complexes were analyzed to Identify local variation in the residues (Figure 4). The RMSF values for all compounds almost remained below 2 Å for most residues, that signifying little atomic fluctuations and a stable overall backbone during the 100 ns simulation. Noteworthy peaks were noted mostly in areas associated with loop segments and terminal residues, specifically at residue indices 30-50, 160-200, and 270-300, which are generally solvent-exposed and facilitate inherent flexibility in the protein. The reference complex (ADP) and TMC-52A exhibited minimal variance across all systems, indicating robust stability in the catalytic and binding-site areas. Reasonable fluctuations were notice in the Fosfocytocin and Molybdopterin Z complexes, particularly at the loop surrounding residues 410-420, a region also implicated in ligand interactions as indicated by the docking studies. These changes probably signify adaptive movements that aid in ligand accommodation rather than causing instability. Compounds Muscimol and 5-amino-2-(3-hydroxy-13-methyltetradecanamido) pentanoic acid showd slightly increased flexibility in distal loops while being within the range documented for proteins undergoing conformational adjustments.

The RMSF pattern consitent with the RMSD results, that showed the ligand binding did not induce any unusual fluctuation. The Flexibility centered around binding pocket residues including Gly413, Gly415, Lys416, Thr417, Phe418, Arg316, Arg318, Pro319, Leu321, Thr412, and Ser414 facilitates small conformational modifications, aligning with previous findings on structural adaptability in kinesin like motor proteins. Additionally, Ligands RMSF data present in Supplementary Figure S3. After evaluating stability and flexibility by RMSD and RMSF analysis, protein-ligand contact mapping was done to the critical residues that facilitate stable interactions throughout the simulation.

### Protein-ligand contact and interaction mapping

**FIGURE 5:**
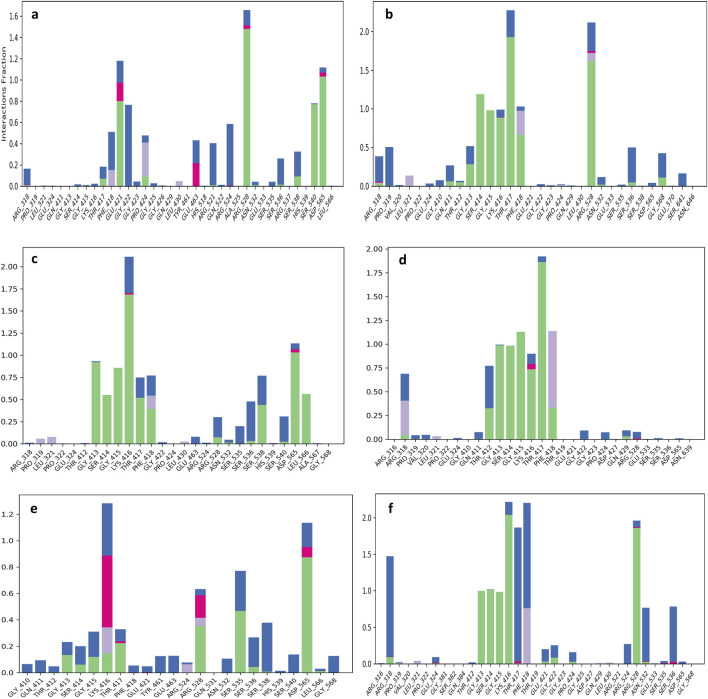
Protein-ligand interaction mapping was conducted for the KIFC1 protein docked with selected natural compounds: **(a)** TMC-52A, **(b)** Fosfocytocin, **(c)** 5-amino-2-(3-hydroxy-13-methyltetradecanamido) pentanoic acid, **(d)** Molybdopterin compound Z, **(e)** Muscimol, and **(f)** the reference compound Adenosine 5′-diphosphate, utilizing data derived from 100 ns molecular dynamics simulations.

**FIGURE 6:**
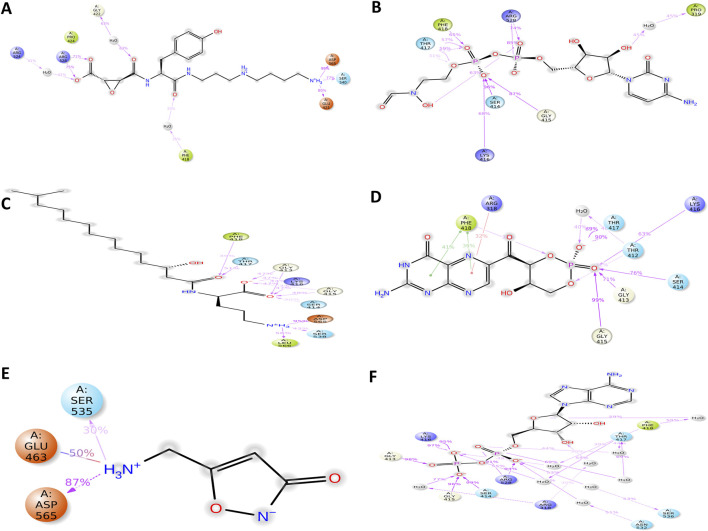
A detailed schematic representation of atomic interactions between the ligands of natural compounds and the reference molecule **(A)** TMC-52A, **(B)** Fosfocytocin, **(C)** 5-amino-2-(3-hydroxy-13-methyltetradecanamido) pentanoic acid, **(D)** Molybdopterin compound Z, **(E)** Muscimol, and **(F)** the reference compound Adenosine 5′-diphosphate docked with the KIFC1 is presented. Based on the selected trajectory (100 ns), only those interactions that occurred for more than 30.0% of the simulation period are displayed.

Protein-ligands complex contact analysis provide as valuable approaches to understanding the structural and functional features of ligand that bind within the active site of proteins. MD simulation over 100 ns provide detailed insight into atomic and intermolecular interactions, which revealing key stabilizing forces like hydrogen bonding, hydrophobic contacts, salt bridges, water-mediated interactions, and electrostatic effects. These interactions have a crucial role in evaluating the conformational stability, binding specificity, and overall affinity of ligands within the protein’s binding pocket. TMC-52A-KIFC1 complex, that have key residues like Gly421, Arg528, and Asp565 were notice as crucial for maintaining ligand alignment and structural stability (Figure 5a). The interaction showed that TMC-52A make 12 hydrogen bonds with eight residues, including strong and persistent bonds with Arg528 and Glu421, which were maintained in approximately 75% and 80% of the simulation frames, correspondingly. Additionally, it was also notice that Gly422 and Phe418 contributed to water-mediated hydrogen bonds, while Asp565 exhibited the highest contact occupancy (99%), highlighting its essential role in ligand stabilization. Furthermore, stabilization was provided by residues Arg524 and Ser540 through both direct and solvent-mediated interactions (Figure 6A).

Fosfocytocin-KIFC1 complex, residues Thr417, Arg528, and Phe418 exhibited the highest interaction frequencies (Figure 5b). Fosfocytocin-KIFC1 compound established 13 hydrogen bonds with 8 key residues, like Ser414, Gly415, Lys416, Thr417, Arg528, and Phe418. Among these, Ser414 and Arg528 displayed the most stable hydrogen bonding interactions, maintained in over 85% of the simulated frames. H2O bridges involving Pro319 and Thr417 enhanced ligand retention within the binding pocket. Although π–π stacking interactions were absent, the ligand formed a dense polar interaction network, leading to a distinctive and highly stable binding configuration (Figure 6B). The 5-amino-2-(3-hydroxy-13-methyltetradecanamido) pentanoic acid KIFC1 complex exhibited a strong and stable interaction within the protein active site. Interaction mapping highlighted Lys416 and Asp565 as key residues, mostly involved in hydrogen bonding and hydrophobic interactions that anchored the ligand firmly in place (Figure 5c). Detailed contact analysis revealed that the ligand formed eleven hydrogen bonds with nine residues-Thr417, Gly413, Lys416, Gly415, Ser414, Asp565, Ser538, Leu566, and Phe418. Among these, Asp565 established a stable salt bridge with the ligand’s ammonium group, persisting through 95% of the simulation frames and underscoring its central role in ligand stability. Water-mediated interactions were also observed with Ser538 (43%) and Leu566 (56%), contributing to solvation-driven stabilization. Moderate but consistent hydrogen bonding occurred with Thr417 (51%), Lys416 (44%–47%), Gly413 (47%), Gly415 (47%), and Ser414 (30%–48%), while Phe418 formed an additional water-mediated contact (39%), strengthening the overall interaction network (Figure 6C). The molybdopterin compound Z-KIFC1 complex displayed a diverse and consistent set of molecular contacts, which played a major role in stabilizing the ligand inside the active site. Interaction mapping identified Gly415, Thr417, and Phe418 as central residues responsible for establishing a mix of polar and nonpolar interactions (Figure 5d). Contact analysis further showed robust and long-lived hydrogen and electrostatic interactions with Gly413, Gly415, Thr412, Thr417, Ser414, and Lys416. Gly415 (99%), Thr417 (90%), Thr412 (89%), and Gly413 (71%) maintained consistent hydrogen bonding with the ligand’s phosphate group, crucial for maintaining its orientation. Additional hydrogen bonds with Ser414 (76%) and Lys416 (63%) enhanced structural stability. Water-mediated contacts with Thr417 and Thr412 (each ∼40%) supported hydration-based stabilization. Phe418 contributed aromatic stability through π–π stacking (41%) and π–cation interactions (36%), while Arg318 participated in electrostatic interactions (32% occupancy), reinforcing the charged interface of the binding pocket (Figure 6D).

Muscimol-KIFC1 complex, Asp565 appeared as the dominant residue, showing the highest interaction occupancy, supported by Arg528 and Ser535 shown in Figure 5e. The ligand’s NH_3_^+^ group formed a strong salt bridge with Asp565 (87%), complemented by electrostatic and hydrogen-bonding interactions that reinforced the binding. Glu463 contributed additional electrostatic and polar stabilization (50%), and Ser535 exhibited polar interactions (30%), that supporting the complex’s structural integrity (Figure 6E). Reference-KIFC1 complex presented a specific interaction pattern defined by hydrophobic and polar hydrogen-bond contacts, illustrating the protein’s flexibility during ligand accommodation. Notably, Thr417 and Thr418 displayed high interaction frequencies (Figure 5f). ADP formed strong interactions with Gly413 (98%), Gly415 (96%), Lys416 (95%), Ser414 (99%), Arg528 (94%), and Arg318 (30%–55%). Hydrogen bonds with Thr417 (44%) and water-mediated contacts (69%, 53%) contributed to solvation and maintained ligand orientation. The adenine ring engaged in both polar and hydrophobic contacts with Phe418 (59%), while Asn532 and Ser536 strengthened the complex through water bridges (53%, 51%) (Figure 6F). Together, these interaction networks highlight a stable set of electrostatic, hydrogen-bonding, water-mediated, and aromatic forces that maintain ligand stability within the KIFC1 binding pocket. These findings provide a strong basis for future inhibitor design targeting KIFC1. Following this mapping analysis, the radius of gyration (Rg) was evaluated to assess the overall compactness and structural stability of the protein during the simulation.

### Analysis of the radius of gyration

Radius of gyration was evaluate to assess the overall compactness and conformational stability of the KIFC1 protein throughout the 100 ns MD simulation with ligands. Rg values indicate the degree of compactness or looseness in a protein’s folding; a lower Rg signifies a more compact and stable structure.

**FIGURE 7:**
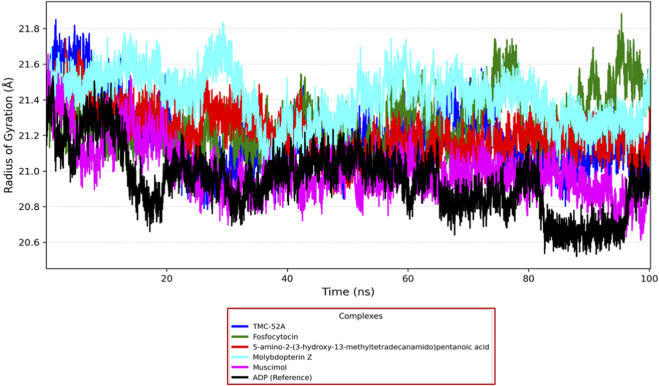
Radius of gyration plots for the KIFC1 protein in complex with the selected natural compounds, represented by the following color scheme: blue (TMC-52A), green (Fosfocytocin), red (5-amino-2-(3-hydroxy-13-methyltetradecanamido) pentanoic acid), cyan (Molybdopterin Z), magenta (Muscimol), and black (adenosine 5′-diphosphate, reference).

The TMC-52A-KIFC1 complex exhibited a progressive decline in Rg from 21.6 Å to 20.8 Å over the interval of 10–60 ns, subsequently stabilizing between 20.8-21.2 Å, which signifies commendable structural tightness. The Fosfocytocin-KIFC1 complex initiated at 21.3-21.4 Å, decreased to around 20.9 Å by 50 ns, and subsequently sustained stability within the range of 21.0–21.3 Å, indicating that the protein remained structurally intact. The 5-amino-2-(3-hydroxy-13-methyltetradecanamido) pentanoic acid-KIFC1 complex exhibited a steady decrease in Rg from 21.5 Å to around 21.0 Å across the 20–60 ns interval, indicating enhanced compactness and stability. The Rg value for the Molybdopterin Z-KIFC1 complex rose from 21.4 Å to 21.8 Å at around 60 ns, indicating a transient expansion resulting from structural reconfiguration, before subsequently declining to 21.1 Å, thereby attaining a stable conformation by the conclusion of the simulation. The Muscimol-KIFC1 complex exhibited the most rapid and pronounced compaction, with Rg decreasing significantly from 21.6 Å to below 21.0 Å during the initial 30 ns, subsequently stabilizing between 20.8-21.0 Å until the conclusion. The ADP reference complex exhibited a comparable stable pattern, with Rg decreasing from 21.2-21.5 Å to below 21.0 Å at around 30 ns, subsequently remaining between 20.8-21.0 Å, signifying the characteristic stability trend of the reference (Figure 7).

The data collectively demonstrate that all evaluated ligands facilitated the conformational compaction of KIFC1 to differing extents, with Muscimol exhibiting the most consistent stabilising effects. The Rg analysis substantiates the capacity of these compounds to enhance the structural integrity and stability of KIFC1 during ligand binding.

### MM/GBSA analysis

A crucial aspect of drug development is optimizing the molecular interactions between the therapeutic candidate and its target. A structural approach alone is insufficient to account for this process. Consequently, multiple interconnected approaches are needed to evaluate the dynamic factors involved in the comprehensive binding process of a ligand to a protein. Consequently, we have conducted thermodynamic measurements Prime MM-GBSA to ascertain the energy of the optimized free receptors, free ligands, and ligand-receptor complexes. MM/GBSA free energy offered a quantitative assessment of the ligand-protein interaction strength.

The net binding free energy of the retrieved poses from the final 10 ns of the 100 ns molecular dynamics simulation trajectory was calculated using the MM/GBSA method. The findings demonstrated that of all examined ligands, Fosfocytocin displayed the highest advantageous binding energy (ΔG_bind = −46.30 kcal/mol), signifying a greater affinity for the KIFC1 protein. Subsequently, 5-amino-2-(3-hydroxy-13-methyltetradecanamido) pentanoic acid exhibited a ΔG_bind of −43.18 kcal/mol, while Molybdopterin Compound Z revealed a modest binding energy of ΔG_bind = −33.68 kcal/mol. TMC-52A (ΔG_bind = −19.52 kcal/mol) and Muscimol (ΔG_bind = −16.04 kcal/mol) exhibited diminished binding affinities relative to the other evaluated ligands. All studied natural compounds had negative free energy values, indicating advantageous interactions with the protein.

The binding free energy of the reference complex ADP-KIFC1 was determined to be −24.52 kcal/mol, while Fosfocytocin, the 5-amino-2-(3-hydroxy-13-methyltetradecanamido) pentanoic acid molecule, and Molybdopterin Z exhibited higher advantageous binding energies. This suggests that these three primary natural compounds may function as more effective binders than the endogenous ligand. Thus, MM/GBSA binding free energy analysis validates the comparative stability and binding efficacy of these natural candidates with the KIFC1 protein, establishing a foundation for their subsequent advancement as potential inhibitors.

**FIGURE 8:**
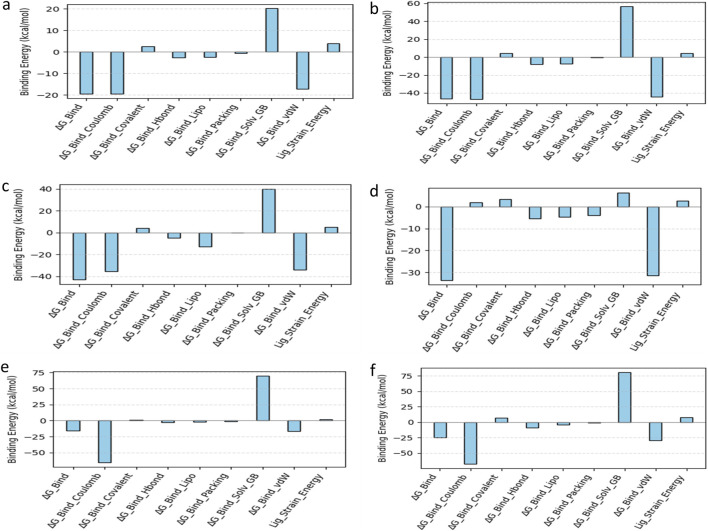
Calculated net binding free energy and energy component values for KIFC1 protein complexes with selected natural compounds: **(a)** TMC-52A, **(b)** Fosfocytocin, **(c)** 5-amino-2-(3-hydroxy-13-methyltetradecanamido) pentanoic acid, **(d)** Molybdopterin compound Z, **(e)** Muscimol, and **(f)** the reference compound Adenosine 5′-diphosphate. The data was derived from snapshots taken from the final 10 ns of the simulation trajectory of the molecular dynamic.

The binding energy decomposition analysis of five ligand and reference compounds-protein complexes demonstrated significant variation in their interaction patterns. All five compounds had a greater binding affinity than the reference compound, as shown by their more advantageous energy contributions (Figure 8). To evaluate the stability of protein-ligand complexes within the binding pocket, we also examined the time-dependent interaction profile during the simulation. In contrast to empirical scoring functions commonly used in docking tools, MM/GBSA shown a higher connection with experimental binding affinities (Supplementary Figure S4).

### Principal component analysis (PCA)

PCA of protein-ligand Complexes was conducted to investigate the fundamental collective motions of the KIFC1 protein in association with selected natural compounds. The analysis was conducted on MD trajectories by computing covariance matrices of backbone Cα atom fluctuations following the alignment of frames to a reference structure to remove translational and rotational movements. The initial two principal components (PC1 and PC2), denoting the most prominent modes of motion, were extracted for comparative study.

**FIGURE 9:**
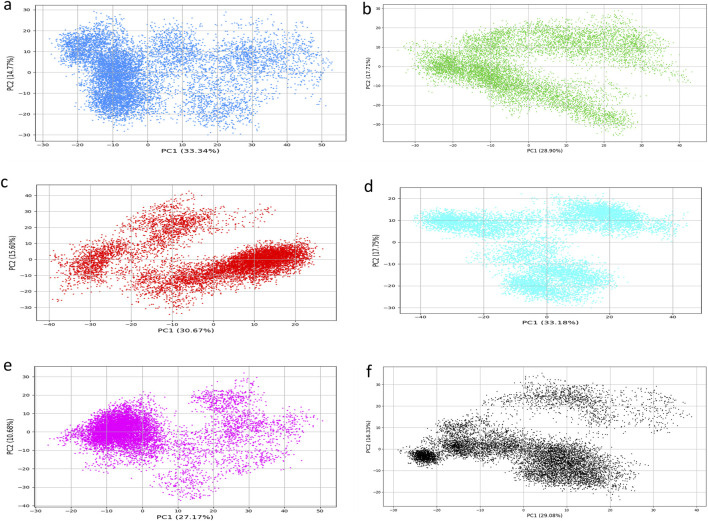
Principal Component Analysis (PCA) of molecular dynamics simulation trajectories for KIFC1 docked with: **(a)** TMC-52A, **(b)** Fosfocytocin, **(c)** 5-amino-2-(3-hydroxy-13-methyltetradecanamido) pentanoic acid, **(d)** Molybdopterin compound Z, **(e)** Muscimol, and **(f)** the reference compound Adenosine 5′-diphosphate.

The TMC-52A-KIFC1 complex exhibited the most conformational, with PC1 and PC2 representing 33.34% and 14.77% of the total motion, respectively, signifying considerable structural flexibility (Figure 9a). In contrast, the Fosfocytocin-KIFC1 complex shown diminished dynamics (PC1 = 28.90%, PC2 = 17.71%) (Figure 9b), whilst the 5-amino-2-(3-hydroxy-13-methyltetradecanamido) pentanoic acid substituted ligand-KIFC1 complex displayed somewhat restricted movements (PC1 = 30.67%, PC2 = 15.60%) (Figure 9c). The Molybdopterin compounds Z-KIFC1 exhibited (PC1 = 33.18%, PC2 = 17.75%), indicating flexible binding or partial destabilization (Figure 9d). Muscimol-KIFC1 exhibited markedly decreased mobility (PC1 = 27.17%, PC2 = 10.68%), signifying a rigidified structure (Figure 9e). The reference compounds ADP-KIFC1 complex exhibited limited dynamics (PC1 = 29.08%, PC2 = 16.33%), aligning with its established function in stability (Figure 9f).

Data demonstrate that these ligands binding substantially alters the conformational landscape of KIFC1 protein. Compounds like Muscimol and Fosfocytocin diminish the protein’s flexibility, potentially limiting its functional dynamics and contributing to inhibition. Conversely, TMC-52A and Molybdopterin Z also demonstrate favorable PCA results, signifying consistent structural behaviour upon binding. These compounds may effectively inhibit KIFC1 by stabilising certain conformations that disrupt its normal activity.

## Discussion

Kinesin motor proteins facilitate cellular function by utilizing energy derived from adenosine triphosphate (ATP). There are fourteen kinesin variants, with a minimum of seven participating in cell division rather than transporting vesicles or organelles within the cell. They all have a similar motor domain that binds to microtubules and breaks down ATP for energy. This segment has unique sequence that are nearly identical in overall kinesin proteins. A modification of a single amino acid within these sequences might influence motor functionality and induce cellular alterations (Park et al., 2017). In a study Wei Hu revealed that the motor protein kinesin operates utilizing energy derived from ATP. Through light microscopy, it was observed that kinesin advances precisely 8.12 nm (nm) along a microtubule with the hydrolysis of each ATP molecule. This verifies that a single ATP molecule facilitates one specific step of kinesin (Hua et al., 1997). Numerous prior studies have validated the critical role of ATP in kinesin activity. Consequently, the aim of our study was to inhibit the ATP active site in the protein with natural compounds, thereby preventing ATP from binding to the site. Without ATP binding and its subsequent hydrolysis, the protein is rendered non-functional. To accomplish this inhibition, we examined a library of natural compounds aimed at the same amino acid residues in the KIFC1 protein that are recognized for binding ADP. The objective of obstructing these critical binding sites was to inhibit ATP’s interaction with the protein, thereby interrupting its function and leaving it inactive.

**FIGURE 10:**
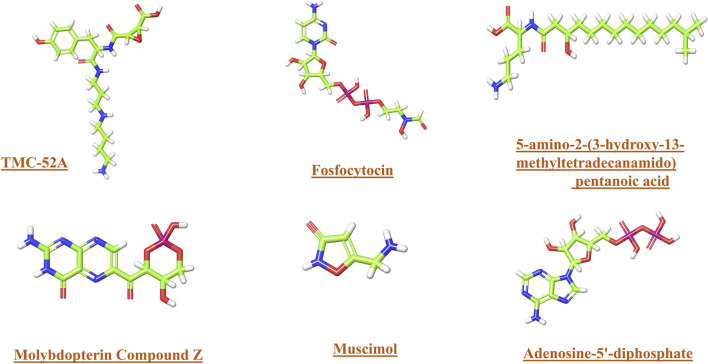
The structure of lead compounds TMC-52A, Fosfocytocin, 5-amino-2-(3-hydroxy-13-methyltetradecanamido) pentanoic acid, Molybdopterin compound Z, Muscimol, and the reference compound Adenosine 5′-diphosphate.

In the present study these compounds like Fosfocytocin exhibited the most robust binding affinity (−46.30 kcal/mol) and substantially inhibited KIFC1 protein mobility, establishing it as the leading candidate. The 5-amino-2-(3-hydroxy-13-methyltetradecanamido) pentanoic acid molecule exhibited robust binding (−43.18 kcal/mol), favourable drug-like characteristics, and persistent interactions, indicating significant promise. TMC-52A had the highest docking score (−7.862), commendable stability, and robust interactions, positioning it as a viable scaffold. Molybdopterin Z and Muscimol exhibited significant interactions, but with reduced binding energies. Common binding residues such as Gly413, Ser414, Lys416, and Arg528 were implicated in the leading compounds. Fosfocytocin, the 5-amino-2-(3-hydroxy-13-methyltetradecanamido) pentanoic acid compound, and TMC-52A emerge as prominent prospects for additional investigation targeting KIFC1 in TNBC. To our knowledge, these compounds have not been documented in previous study concerning KIFC1 inhibition. Prior finding has predominantly concentrated on synthetic inhibitors or established natural compounds, but our results uncover unexplored natural compounds with significant binding affinity Figure 10 shows the structure of these compounds.

In a recent study, Muhammad Bilal Azmi et al. investigated the anticancer properties of propolis, a resinous substance generated by bees, emphasizing its capacity to inhibit the KIFC1, utilizing *in silico* approach, they identified five compounds derived from propolis: Kaempferide (ΔG = −7.35 kcal/mol), Luteolin (ΔG = −6.74 kcal/mol), Izalpinin (ΔG = −6.33 kcal/mol), 4′,5,7-Trihydroxy-3,6-dimethoxyflavone (ΔG = −6.14 kcal/mol), and 6-Methoxykaempferol (ΔG = −6.55 kcal/mol). These compounds exhibited advantageous interactions with the active binding sites of KIFC1. To further their validation, 100-ns molecular dynamics (MD) simulations were performed. Of the five compounds, 4′,5,7-trihydroxy-3,6-dimethoxyflavone and 6-methoxykaempferol had notably stable and substantial interactions with essential KIFC1 residues during the simulation period, hence affirming their binding stability (Azmi et al., 2025). In contrast to their findings, us identify compounds demonstrate enhanced binding affinities, suggesting increased inhibitory ability against KIFC1 and underscoring their potential as novel, natural options for TNBC treatment. AZ82 is the first documented inhibitor that selectively targets the KIFC1 (Sharma et al., 2023). It directly binds to the KIFC1-microtubule complex and inhibits KIFC1 activity in an ATP-competitive, exhibiting a potent inhibition constant (Ki) of 0.043 μM. The cellular effects highlight the therapeutic significance of KIFC1; study suggest that inhibition of KIFC1 by AZ82 is a viable approach for anticancer medication development (Wu et al., 2013). The lead compounds notice in this study were selectively evaluated for competitive inhibition and demonstrated significant and promising inhibitory efficacy. Similarly, Wei Zhang and colleagues identify SR31527 that significantly suppressed KIFC1 ATPase activity, with an IC_50_ value of 6.6 μM. Bio-layer interferometry demonstrated that the drug binds directly to KIFC1 with high affinity (Kd = 25.4 nM) (Zhang et al., 2016).

As previously mentioned, while earlier research by Azmi et al. (2025), Wu et al. (2013), and Zhang et al. (2016) has examined synthetic and propolis-derived KIFC1 inhibitors, the present study is unique in its identification of novel natural compounds from the Natural Products Atlas via a thorough *in silico* methodology. The chosen molecules-TMC-52A, Fosfocytocin, 5-amino-2-(3-hydroxy-13-methyltetradecanamido) pentanoic acid, Molybdopterin Compound Z, and Muscimol-have not been reported in context to KIFC1 inhibition or therapy for TNBC. TMC-52A has been identified as a cysteine protease inhibitor (Isshiki et al., 1998), while Muscimol functions as a GABAA receptor agonist (Johnston, 2014), however, the remaining compounds have no previous biological documentation concerning cancer or KIFC1. This extensive chemical investigation and comprehensive computational validation highlight the originality of our findings and establish a new paradigm for identifying natural KIFC1 inhibitors pertinent to TNBC treatment.

The comprehensive computational evaluations underscore the efficacy of the chosen drugs as KIFC1 inhibitors (Boehm et al., 2000). Docking results demonstrated robust binding affinities and stable contacts with critical residues in the ATP-binding pocket, indicating a potential inhibition of KIFC1’s motor function vital for centrosome clustering and spindle organization in cancer cells (Park et al., 2017). ADMET and toxicity assessments corroborated the drug-likeness and safety of these compounds, suggesting advantageous pharmacokinetic characteristics (Banerjee et al., 2018). DFT study further validated their electronic stability and reactivity, which are crucial for biological activity and target interaction (Siegbahn and Blomberg, 1999). Molecular dynamics simulations revealed stable protein-ligand complexes characterized by modest RMSD fluctuations, consistent RMSF and Rg profiles, and the retention of ligands within the binding pocket throughout the trajectory (Liu and Kokubo, 2017). Collectively, these findings indicate that the selected compounds exhibit the requisite structural and energetic properties for KIFC1 inhibition and may represent viable candidates for experimental validation in cancer therapies.

## Conclusion

This study intended to investigate natural compounds that can inhibit KIFC1, a protein that potentially participate in proliferation and survival of BC, particularly in TNBC. Employing sophisticated computational methods, we evaluated thousands of natural compounds and successfully found five compounds with significant potential to inhibit the function of KIFC1. Among them Fosfocytocin exhibited the most capacity to inhibit KIFC1, establishing itself as the most promising compounds. The Molybdopterin compounds Z exhibited substantial inhibitory efficacy, ranking as the second most effective molecule in our study. The remaining compounds 5-amino-2-(3-hydroxy-13-methyltetradecanamido) pentanoic acid, TMC-52A, and Muscimol demonstrated favourable results, with acceptable value and stable interactions with the target protein.

Our findings indicate that the natural compounds Fosfocytocin and Molybdopterin Compound may be promising candidates for KIFC1-targeted treatments in TNBC. However, additional *in vitro* and *in vivo* validation is required to ascertain their therapeutic efficacy and safety before to progressing to clinical applications.

## Data Availability

The raw data supporting the conclusions of this article will be made available by the authors, without undue reservation.
