# Supplementary material for: In silico identification of novel natural compounds as potential KIFC1 inhibitors for the therapeutic intervention of triple-negative breast cancer
Source: Front Bioinform. 2025 Dec 16;5:1689172. doi: 10.3389/fbinf.2025.1689172 (PMC12748000; doi:10.3389/fbinf.2025.1689172)
Supplement: Supplementary file 1 [file Supplementaryfile1.docx]

**Supplementary File**

**supplementary Table S1.** List of top Twelve Structure-based virtual screening (SBVS) natural compounds against KIFC1 protein with their result.

| npaid | compound_name | docking score | glide energy |
| --- | --- | --- | --- |
| NPA009447 | TMC-52A | -7.862 | -60.913 |
| NPA021320 | Fosfocytocin | -7.857 | -57.934 |
| NPA034881 | 5-amino-2-(3-hydroxy-13-methyltetradecanamido) pentanoic acid | -7.687 | -53.292 |
| NPA016275 | Molybdopterin compound Z | -7.384 | -45.513 |
| NPA024780 | Muscimol | -7.250 | -24.679 |
| NPA035922 | 12β-DeoxyGTX5 | -7.234 | -48.051 |
| NPA032650 | AAL Toxin TC2 | -7.224 | -53.070 |
| NPA024004 | Aspergillide E | -7.085 | -56.643 |
| NPA015131 | Amphistin | -7.084 | -45.370 |
| NPA008605 | Sulphostin | -7.031 | -43.287 |
| NPA003384 | Baeocystin | -7.026 | -39.943 |
| NPA034882 | 5-amino-2-(3-hydroxy-14-methylpentadecanamido) pentanoic acid | -7.014 | -48.699 |

**supplementary Table S2**: ADME analysis for the top five selected natural compounds as inhibitor against KIFC1.

| **Properties** | **TMC-52A** | **Fosfocytocin** | **5-amino-2-(3-hydroxy-13-methyltetradecanamido)pentanoic acid** | **Molybdopterin Compound Z** | **Muscimol** |
| --- | --- | --- | --- | --- | --- |
| Formula | C20H30N4O6 | C12H20N4O13P2 | C20H40N2O4 | C10H10N5O7P | C4H6N2O2 |
| MW | 422.48 | 490.25 | 372.54 | 343.19 | 114.1 |
| Heavy atoms | 30 | 31 | 26 | 23 | 8 |
| #Aromatic heavy atoms | 6 | 6 | 0 | 10 | 5 |
| Fraction Csp3 | 0.55 | 0.58 | 0.9 | 0.3 | 0.25 |
| Rotatable bonds | 16 | 11 | 18 | 2 | 1 |
| H-bond acceptors | 8 | 14 | 5 | 10 | 3 |
| H-bond donors | 6 | 6 | 4 | 4 | 2 |
| MR | 108.18 | 96.23 | 106.9 | 73.21 | 27 |
| TPSA | 166.31 | 273.05 | 112.65 | 200.42 | 72.02 |
| iLOGP | 2.24 | -0.69 | 3.39 | -0.67 | 0.82 |
| XLOGP3 | -2.74 | -4.96 | 1.82 | -2.81 | -2.39 |
| WLOGP | -0.89 | -2.77 | 3.21 | -1.64 | -0.73 |
| MLOGP | -1.15 | -3.55 | 2.06 | -2.85 | -1.08 |
| Silicos-IT Log P | 1.07 | -5.29 | 3.98 | -1.17 | 0.67 |
| CLog P | -0.3 | -3.45 | 2.89 | -1.83 | -0.54 |
| ESOL Log S | 0.17 | 0.83 | -2.11 | -0.39 | 0.56 |
| ESOL Solubility (mg/ml) | 6.32E+02 | 3.30E+03 | 2.90E+00 | 1.41E+02 | 4.16E+02 |
| ESOL Solubility (mol/l) | 1.50E+00 | 6.73E+00 | 7.79E-03 | 4.10E-01 | 3.65E+00 |
| ESOL Class | Highly soluble | Highly soluble | Soluble | Very soluble | Highly soluble |
| Ali Log S | -0.2 | -0.14 | -3.81 | -0.84 | 1.42 |
| Ali Solubility (mg/ml) | 2.66E+02 | 3.57E+02 | 5.83E-02 | 4.91E+01 | 2.98E+03 |
| Ali Solubility (mol/l) | 6.30E-01 | 7.27E-01 | 1.57E-04 | 1.43E-01 | 2.61E+01 |
| Ali Class | Very soluble | Very soluble | Soluble | Very soluble | Highly soluble |
| Silicos-IT LogSw | -3.91 | 2.98 | -4.45 | -0.76 | -1.13 |
| Silicos-IT Solubility (mg/ml) | 5.21E-02 | 4.70E+05 | 1.33E-02 | 5.91E+01 | 8.38E+00 |
| Silicos-IT Solubility (mol/l) | 1.23E-04 | 9.59E+02 | 3.57E-05 | 1.72E-01 | 7.35E-02 |
| Silicos-IT class | Soluble | Soluble | Moderately soluble | Soluble | Soluble |
| GI absorption | Low | Low | High | Low | High |
| BBB permeant | No | No | No | No | No |
| Pgp substrate | Yes | Yes | Yes | No | No |
| CYP1A2 inhibitor | No | No | No | No | No |
| CYP2C19 inhibitor | No | No | No | No | No |
| CYP2C9 inhibitor | No | No | No | No | No |
| CYP2D6 inhibitor | No | No | No | No | No |
| CYP3A4 inhibitor | No | No | No | No | No |
| log Kp (cm/s) | -10.82 | -12.81 | -7.28 | -10.39 | -8.69 |
| Lipinski violations | 1 | 2 | 0 | 1 | 0 |
| Ghose violations | 1 | 2 | 0 | 1 | 4 |
| Veber violations | 2 | 2 | 1 | 1 | 0 |
| Egan violations | 1 | 1 | 0 | 1 | 0 |
| Muegge violations | 4 | 4 | 1 | 2 | 3 |
| Bioavailability Score | 0.55 | 0.11 | 0.55 | 0.11 | 0.55 |
| PAINS #alerts | 0 | 0 | 0 | 0 | 0 |
| Brenk #alerts | 1 | 4 | 0 | 1 | 0 |
| Leadlikeness violations | 2 | 2 | 2 | 0 | 1 |
| Synthetic Accessibility | 4.17 | 5.21 | 3.93 | 3.92 | 2.23 |

**supplementary Table S3:** Toxicity analysis for the top five selected natural compounds as inhibitor against KIFC1.

**TMC-52A**

| **Classification** | **Target** | **Shorthand** | **Prediction** | **Probability** |
| --- | --- | --- | --- | --- |
| Organ toxicity | Hepatotoxicity | dili | Inactive | 0.86 |
| Organ toxicity | Neurotoxicity | neuro | Inactive | 0.54 |
| Organ toxicity | Nephrotoxicity | nephro | Active | 0.68 |
| Organ toxicity | Respiratory toxicity | respi | Active | 0.77 |
| Organ toxicity | Cardiotoxicity | cardio | Active | 0.58 |
| Toxicity end points | Carcinogenicity | carcino | Inactive | 0.63 |
| Toxicity end points | Immunotoxicity | immuno | Inactive | 0.95 |
| Toxicity end points | Mutagenicity | mutagen | Active | 0.55 |
| Toxicity end points | Cytotoxicity | cyto | Inactive | 0.70 |
| Toxicity end points | BBB-barrier | bbb | Inactive | 0.78 |
| Toxicity end points | Ecotoxicity | eco | Inactive | 0.62 |
| Toxicity end points | Clinical toxicity | clinical | Active | 0.64 |
| Toxicity end points | Nutritional toxicity | nutri | Inactive | 0.56 |
| Metabolism | Cytochrome CYP1A2 | CYP1A2 | Inactive | 0.96 |
| Metabolism | Cytochrome CYP2C19 | CYP2C19 | Inactive | 0.93 |
| Metabolism | Cytochrome CYP2C9 | CYP2C9 | Inactive | 0.86 |
| Metabolism | Cytochrome CYP2D6 | CYP2D6 | Inactive | 0.76 |
| Metabolism | Cytochrome CYP3A4 | CYP3A4 | Inactive | 0.93 |
| Metabolism | Cytochrome CYP2E1 | CYP2E1 | Inactive | 0.99 |
| **Fosfocytocin** | | | | |
| Organ toxicity | Hepatotoxicity | dili | Active | 0.69 |
| Organ toxicity | Neurotoxicity | neuro | Active | 0.87 |
| Organ toxicity | Nephrotoxicity | nephro | Inactive | 0.90 |
| Organ toxicity | Respiratory toxicity | respi | Active | 0.98 |
| Organ toxicity | Cardiotoxicity | cardio | Inactive | 0.77 |
| Toxicity end points | Carcinogenicity | carcino | Inactive | 0.62 |
| Toxicity end points | Immunotoxicity | immuno | Active | 0.96 |
| Toxicity end points | Mutagenicity | mutagen | Inactive | 0.97 |
| Toxicity end points | Cytotoxicity | cyto | Inactive | 0.93 |
| Toxicity end points | BBB-barrier | bbb | Inactive | 1 |
| Toxicity end points | Ecotoxicity | eco | Active | 0.73 |
| Toxicity end points | Clinical toxicity | clinical | Inactive | 0.56 |
| Toxicity end points | Nutritional toxicity | nutri | Inactive | 0.74 |
| Metabolism | Cytochrome CYP1A2 | CYP1A2 | Inactive | 0.76 |
| Metabolism | Cytochrome CYP2C19 | CYP2C19 | Inactive | 0.87 |
| Metabolism | Cytochrome CYP2C9 | CYP2C9 | Active | 0.56 |
| Metabolism | Cytochrome CYP2D6 | CYP2D6 | Inactive | 0.63 |
| Metabolism | Cytochrome CYP3A4 | CYP3A4 | Active | 0.71 |
| Metabolism | Cytochrome CYP2E1 | CYP2E1 | Inactive | 0.98 |
| **5-amino-2-(3-hydroxy-13-methyltetradecanamido) pentanoic acid** | | | | |
| Organ toxicity | Hepatotoxicity | dili | Active | 0.69 |
| Organ toxicity | Neurotoxicity | neuro | Active | 0.87 |
| Organ toxicity | Nephrotoxicity | nephro | Inactive | 0.9 |
| Organ toxicity | Respiratory toxicity | respi | Active | 0.98 |
| Organ toxicity | Cardiotoxicity | cardio | Inactive | 0.77 |
| Toxicity end points | Carcinogenicity | carcino | Inactive | 0.62 |
| Toxicity end points | Immunotoxicity | immuno | Active | 0.96 |
| Toxicity end points | Mutagenicity | mutagen | Inactive | 0.97 |
| Toxicity end points | Cytotoxicity | cyto | Inactive | 0.93 |
| Toxicity end points | BBB-barrier | bbb | Inactive | 1 |
| Toxicity end points | Ecotoxicity | eco | Active | 0.73 |
| Toxicity end points | Clinical toxicity | clinical | Inactive | 0.56 |
| Toxicity end points | Nutritional toxicity | nutri | Inactive | 0.74 |
| Metabolism | Cytochrome CYP2C9 | CYP2C9 | Active | 0.56 |
| Metabolism | Cytochrome CYP2D6 | CYP2D6 | Inactive | 0.63 |
| Metabolism | Cytochrome CYP3A4 | CYP3A4 | Active | 0.71 |
| Metabolism | Cytochrome CYP2E1 | CYP2E1 | Inactive | 0.98 |
| **Molybdopterin compound Z** | | | | |
| Organ toxicity | Hepatotoxicity | dili | Active | 0.69 |
| Organ toxicity | Neurotoxicity | neuro | Active | 0.87 |
| Organ toxicity | Nephrotoxicity | nephro | Inactive | 0.9 |
| Organ toxicity | Respiratory toxicity | respi | Active | 0.98 |
| Organ toxicity | Cardiotoxicity | cardio | Inactive | 0.77 |
| Toxicity end points | Carcinogenicity | carcino | Inactive | 0.62 |
| Toxicity end points | Immunotoxicity | immuno | Active | 0.96 |
| Toxicity end points | Mutagenicity | mutagen | Inactive | 0.97 |
| Toxicity end points | Cytotoxicity | cyto | Inactive | 0.93 |
| Toxicity end points | BBB-barrier | bbb | Inactive | 1 |
| Toxicity end points | Ecotoxicity | eco | Active | 0.73 |
| Toxicity end points | Clinical toxicity | clinical | Inactive | 0.56 |
| Toxicity end points | Nutritional toxicity | nutri | Inactive | 0.74 |
| Metabolism | Cytochrome CYP1A2 | CYP1A2 | Inactive | 0.76 |
| Metabolism | Cytochrome CYP2C19 | CYP2C19 | Inactive | 0.87 |
| Metabolism | Cytochrome CYP2C9 | CYP2C9 | Active | 0.56 |
| Metabolism | Cytochrome CYP2D6 | CYP2D6 | Inactive | 0.63 |
| Metabolism | Cytochrome CYP3A4 | CYP3A4 | Active | 0.71 |
| Metabolism | Cytochrome CYP2E1 | CYP2E1 | Inactive | 0.98 |
| **Muscimol** | | | | |
| Organ toxicity | Hepatotoxicity | dili | Active | 0.69 |
| Organ toxicity | Neurotoxicity | neuro | Active | 0.87 |
| Organ toxicity | Nephrotoxicity | nephro | Inactive | 0.9 |
| Organ toxicity | Respiratory toxicity | respi | Active | 0.98 |
| Organ toxicity | Cardiotoxicity | cardio | Inactive | 0.77 |
| Toxicity end points | Carcinogenicity | carcino | Inactive | 0.62 |
| Toxicity end points | Immunotoxicity | immuno | Active | 0.96 |
| Toxicity end points | Mutagenicity | mutagen | Inactive | 0.97 |
| Toxicity end points | Cytotoxicity | cyto | Inactive | 0.93 |
| Toxicity end points | BBB-barrier | bbb | Inactive | 1 |
| Toxicity end points | Ecotoxicity | eco | Active | 0.73 |
| Toxicity end points | Clinical toxicity | clinical | Inactive | 0.56 |
| Toxicity end points | Nutritional toxicity | nutri | Inactive | 0.74 |
| Metabolism | Cytochrome CYP1A2 | CYP1A2 | Inactive | 0.76 |
| Metabolism | Cytochrome CYP2C19 | CYP2C19 | Inactive | 0.87 |
| Metabolism | Cytochrome CYP2C9 | CYP2C9 | Active | 0.56 |
| Metabolism | Cytochrome CYP2D6 | CYP2D6 | Inactive | 0.63 |
| Metabolism | Cytochrome CYP3A4 | CYP3A4 | Active | 0.71 |
| Metabolism | Cytochrome CYP2E1 | CYP2E1 | Inactive | 0.98 |

**
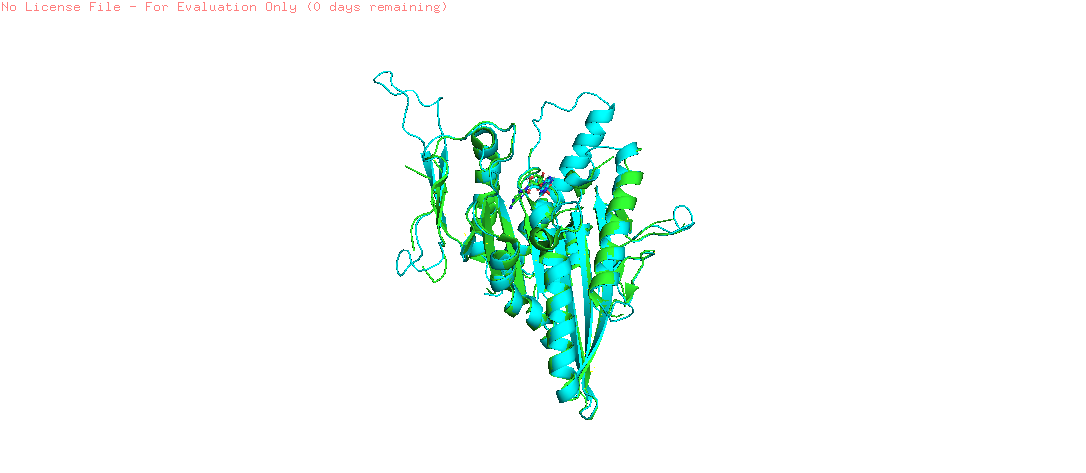
**

**supplementary Figure S1-** Structural superimposition of the native protein–ligand complex and the re-docked complex.The cartoon representation shows the comparison between the native complex (green) and the re-docked complex (cyan). The ligand, visualized in stick representation, is shown to occupy the same binding pocket in both complexes. This structural overlap confirms that the docking protocol successfully reproduced the native binding pose of the ligand, indicating accurate prediction of binding conformation.


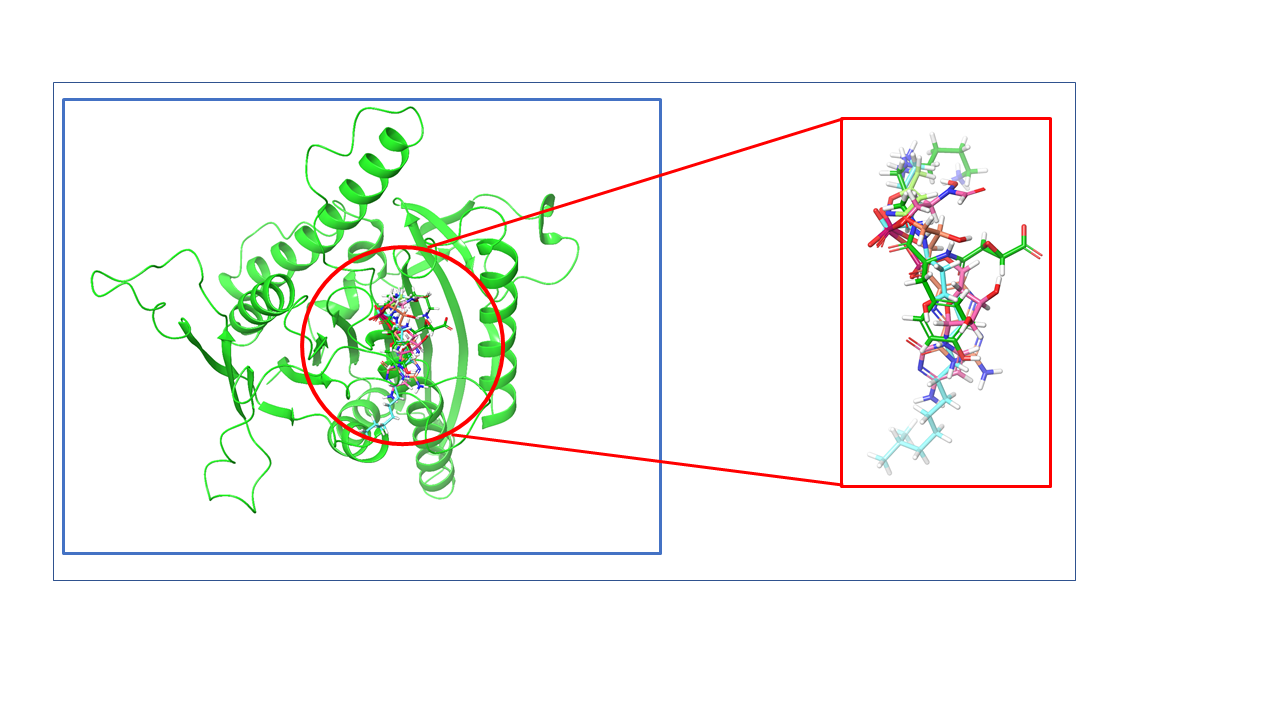


**supplementary Figure S2-**The left panel displays the cartoon representation of the reference protein–ligand complex, with docked compounds shown in stick format at the binding site. The highlighted region indicates the binding pocket. The right panel presents a magnified view of the binding site, showing the superimposition of five docked ligands with the native ligand. The close overlap of these compounds suggests similar binding modes and supports the reliability of the docking protocol.


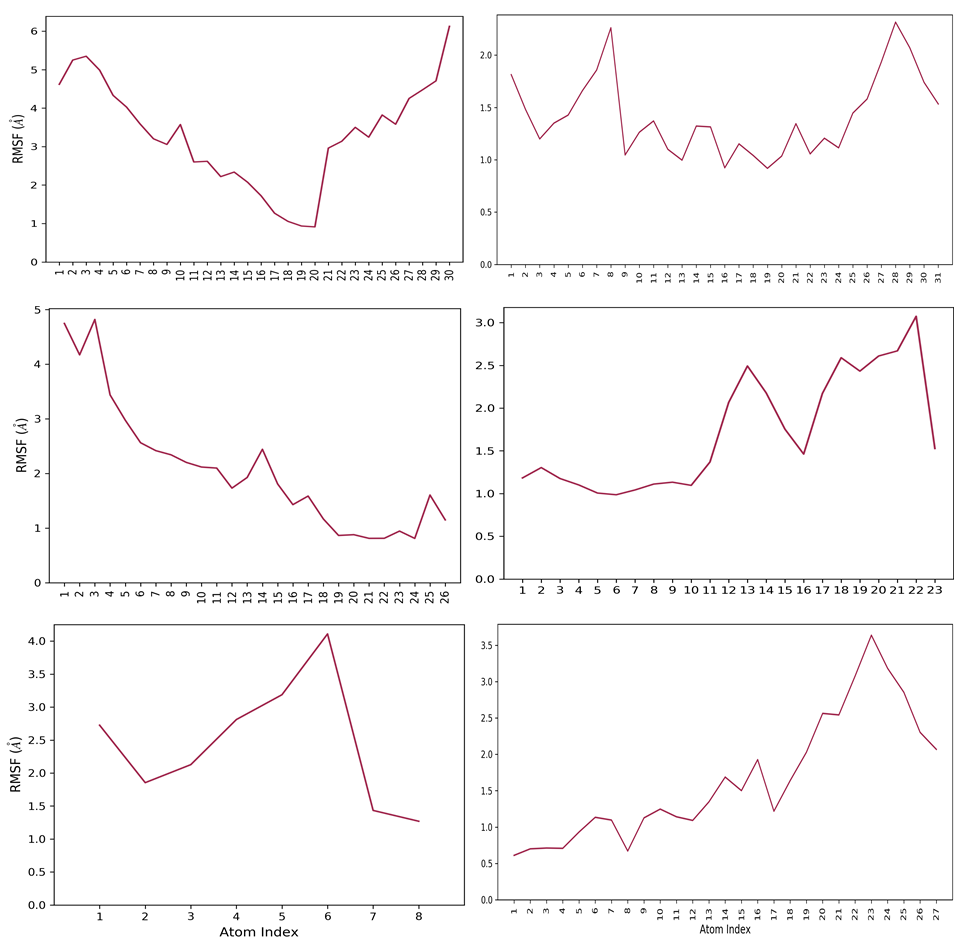


**supplementary Figure S3-** RMSF plots were generated for the docked natural compounds: (A) TMC-52A, (B) Fosfocytocin, (C) 5-amino-2-(3-hydroxy-13-methyltetradecanamido) pentanoic acid, (D) Molybdopterin compound Z, (E) Muscimol, and (F) the reference compound Adenosine 5'-diphosphate, all fitted into the KIFC1 protein during a 100 ns molecular dynamics simulation period.


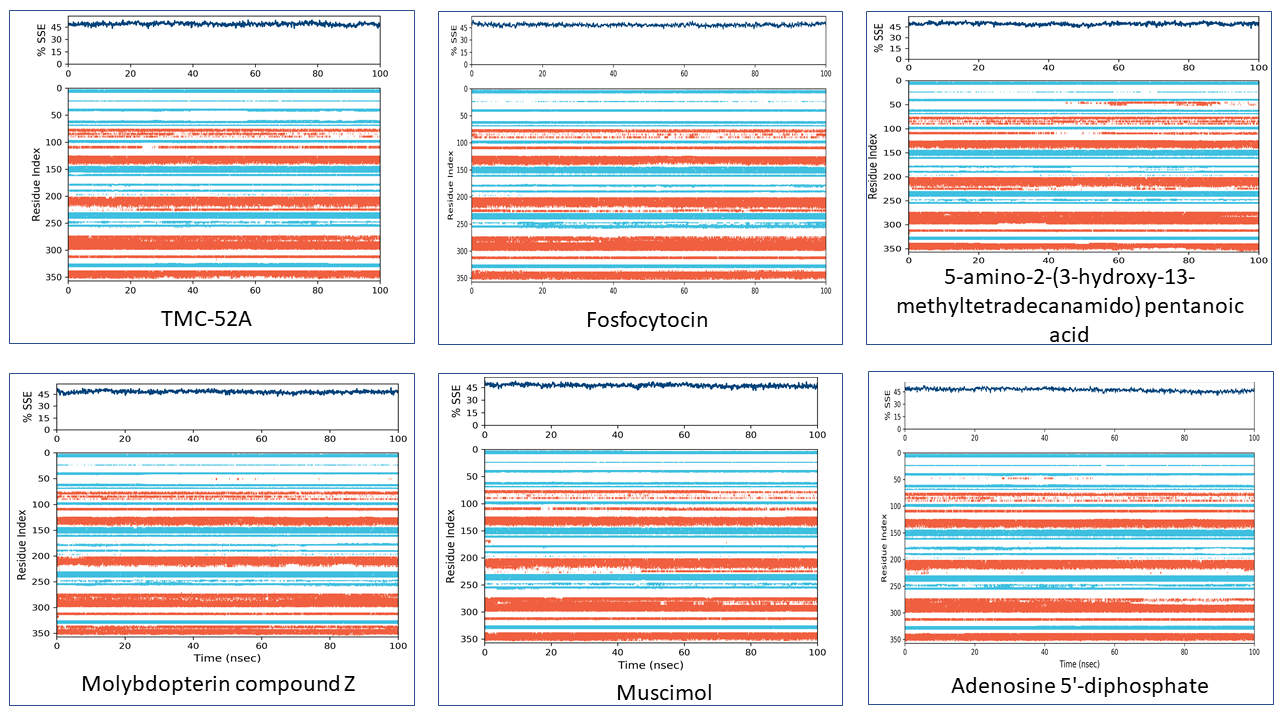


**Figure S4.** A timeline representation of interactions and contacts. The top panel displays total specific contacts between protein and ligand over the whole MD simulation trajectory. The bottom panel indicates residues interacting with the ligand in each frame, with darker shades representing multiple contacts.
